# Identification of novel functional CpG-SNPs associated with Type 2 diabetes and birth weight

**DOI:** 10.18632/aging.202828

**Published:** 2021-04-04

**Authors:** Rui-Ke Liu, Xu Lin, Zun Wang, Jonathan Greenbaum, Chuan Qiu, Chun-Ping Zeng, Yong-Yao Zhu, Jie Shen, Hong-Wen Deng

**Affiliations:** 1 Department of Endocrinology and Metabolism, The Third Affiliated Hospital of Southern Medical University, Guangzhou 510630, China; 2 Department of Endocrinology and Metabolism, SSL Central Hospital of Dongguan City, Dongguan 523326, China; 3 Xiangya Nursing School, Central South University, Changsha 410013, China; 4 Tulane Center for Biomedical Informatics and Genomics, Deming Department of Medicine, School of Medicine, Tulane University, New Orleans, LA 70112, USA; 5 Department of Endocrinology and metabolism, The Fifth Affiliated Hospital of Guangzhou Medical University, Guangzhou 510330, China; 6 School of Basic Medical Sciences, Central South University, Changsha 410000, China

**Keywords:** type 2 diabetes (T2D), birth weight (BW), genome-wide association study (GWAS), DNA methylation, CpG-SNP, cFDR

## Abstract

Genome-wide association studies (GWASs) have identified hundreds of genetic loci for type 2 diabetes (T2D) and birth weight (BW); however, a large proportion of the total trait heritability remains unexplained. The previous studies were generally focused on individual traits and largely failed to identify the majority of the variants that play key functional roles in the etiology of the disease. Here, we aim to identify novel functional loci for T2D, BW and the pleiotropic variants shared between them by performing a targeted conditional false discovery rate (cFDR) analysis that integrates two independent GWASs with summary statistics for T2D (*n* = 26,676 cases and 132,532 controls) and BW (*n* = 153,781) which entails greater statistical power than individual trait analyses. In this analysis, we considered CpG-SNPs, which are SNPs that may influence DNA methylation status, and are therefore considered to be functionally important. We identified 103 novel CpG-SNPs for T2D, 182 novel CpG-SNPs for BW (cFDR < 0.05), and 52 novel pleiotropic loci for both (conjunction cFDR [ccFDR] < 0.05). Among the identified novel CpG-SNPs, 33 were annotated as methylation quantitative trait loci (meQTLs) in whole blood, and 145 displayed at least some effects on meQTL, metabolic QTL (metaQTL), and/or expression QTL (eQTL). These findings may provide further insights into the shared biological mechanisms and functional genetic determinants that overlap between T2D and BW, thereby providing novel potential targets for treatment/intervention development.

## INTRODUCTION

Type 2 diabetes (T2D) is a common chronic metabolic disorder characterized by hyperglycemia, insulin resistance, and impaired insulin secretion [1] It is estimated that in 2017 there were 451 million people suffering from diabetes worldwide [2] 90% of which were classified as T2D [3] Due to the reduced quality of life, increased mortality, and a significant burden on the healthcare system, T2D represents a severe global public health concern. Therefore, it is imperative to gain a better understanding of the pathophysiological mechanisms involved in the onset of T2D for the enhanced development of intervention/treatment strategies.

Birth weight (BW) is a clinical indicator of a variety of metabolic conditions that manifest with age. Studies have demonstrated that low BW is associated with the increased risk of developing T2D [4, 5]. The concept of "developmental programming" holds that events occurring during the early development of an individual and specifically during intrauterine life have profound consequences on future disease such as diabetes [6, 7]. Both T2D and BW are believed to be highly influenced by genetic factors with heritability estimates of over 50% and 37%, respectively [8, 9]. Additionally, the phenotypic correlation between T2D and BW suggests that these traits may share overlapping genetic determinants [10]. Several studies have also proposed the genetic correlation between BW and T2D. For instance, a Mendelian randomization study demonstrated the genetic effects on retarded fetal growth and increased diabetes risk by showing that lower birth weight was associated with increased risk of T2D and higher fasting glucose concentration [11]. Our previous study also identified several loci that associated with both T2D and BW [12]. Therefore, studying genetic relationships between BW and T2D could yield insights into the genetic regulation of T2D risk during the fetal stage.

Hundreds of single nucleotide polymorphisms (SNPs) associated with T2D or BW have been identified by genome-wide association studies (GWASs) [13, 14]. However, these SNPs can only explain a small proportion of the total heritability for T2D (~10%) [15] and BW (~25%) [16]. The large majority of the missing heritability is likely attributed to the well-documented limitations of the single-trait GWAS analysis [17]. Due to the polygenic architecture of most complex traits, many SNPs have associations too weak to be identified with the relatively small sample sizes of current GWASs [18]. Therefore, it is essential to employ statistical approaches that can increase the effective sample size by incorporating more information embedded in the existing univariate datasets. For example, by incorporating the pleiotropic effects among correlated traits, it may be possible to augment the sample sizes of standard GWAS for individual traits and identify more trait-associated loci that would otherwise be missed.

Previous studies suggest that epigenetic mechanisms, which are a crucial link between the genetic factors and environmental exposures [19], may also account for some of the missing heritability [20]. DNA methylation occurs mainly at the fifth position of the cytosine ring in CpG dinucleotides [21], and SNPs that are associated with methylation status are commonly referred to as CpG-SNPs. Although it was previously believed that methylation of the promoter region is responsible for transcriptional silencing, emerging evidence suggests that DNA methylation is closely related to the expression across all genomic regions [19]. DNA methylation is the most well-explored epigenetic mark and is also involved in T2D pathogenesis [22]. For instance, Ma *et al*. [23] identified 30 CpGs representing the whole blood DNA methylation signatures that are associated with cardiovascular disease risk factors and all-cause mortality. Our previous work also proposed that peripheral blood-derived meQTL loci were related to the increasing risk of diabetes and coronary artery disease [23, 24]. Focusing on these CpG-SNPs may be an effective strategy to improve the detection of novel potential functional variants associated with T2D and BW.

A genetic-pleiotropy-informed conditional false discovery rate (cFDR) method was developed to improve the gene discovery in complex traits by integrating two independent GWASs for related traits [25]. A major advantage of this approach is that it only requires the summary statistics rather than the individual level genotype data, which are usually not easily accessible, as well as improves statistical power for identifying novel polygenic effects [25]. Using this approach, our group has analyzed multiple sets of genetically associated traits and successfully identified many novel trait-associated loci [12, 26].

In this study, we applied a targeted cFDR analysis on CpG-SNPs for T2D and BW [13, 14] to identify novel functional loci that are shared between these two traits. Our findings will provide novel insights into the shared pathophysiology between T2D and BW.

## RESULTS

### Assessment of pleiotropic enrichment

**Figure 1:**
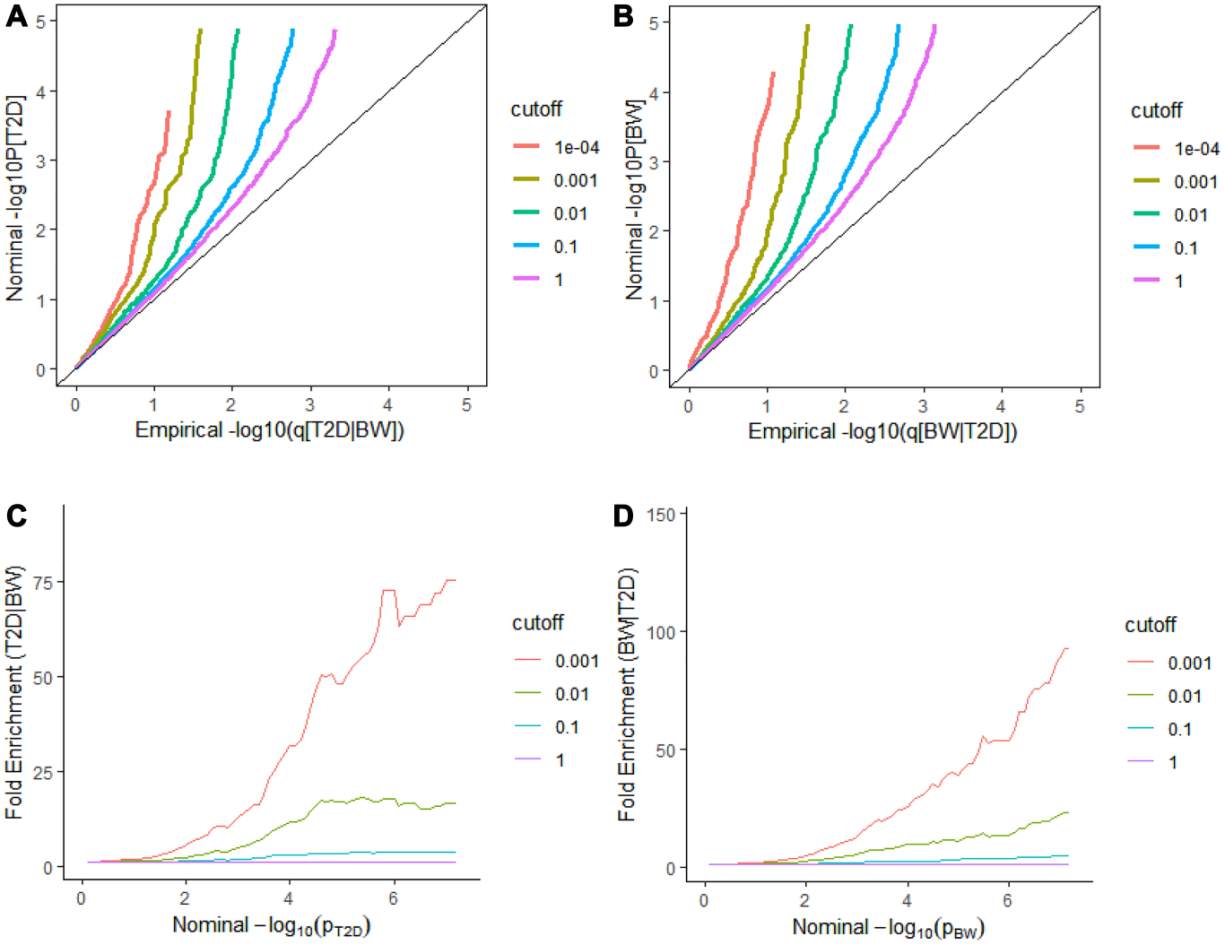
**Stratified Q-Q plots and fold-enrichment plots.** Stratified Q-Q plots of nominal versus empirical -log10(*p*) values in T2D (**A**) as a function of the significance of the association with BW at the level of -log10(*p*) > 0, -log10(*p*) > 1, -log10(*p*) > 2, -log10(*p*) > 3 corresponding to *p* ≤ 1, *p* ≤ 0.1, *p* ≤ 0.01, *p* ≤ 0.001, and *p* ≤ 0.0001, respectively. and (**B**) reversely BW as a function of the significance of the association with T2D. Fold-enrichment plots of enrichment versus nominal -log10p-values (corrected for inflation) corresponding to levels of *p* ≤ 1, *p* ≤ 0.1, *p* ≤ 0.01, *p* ≤ 0.001, respectively in (**C**) T2D as a function of significance of the association with BW; and in (**D**) BW as a function of significance with T2D. Dashed lines indicate the null-hypothesis.

We observed a clear separation between each curve in the conditional Q-Q plot, and the enrichment of effects in T2D varied on different levels of association for BW (Figure 1A and 1C). We can intuitively observe and graphically assess a strong enrichment of T2D-associated SNPs conditioning on various strengths of associations with the BW. The similar separation between the different curves and similar enrichment pattern was also observed in BW conditioned on T2D (Figure 1B and 1D). This result indicates a strong pleiotropy between T2D and BW, regardless of whether T2D is conditioned on BW or BW is conditioned on T2D.

### T2D loci identified by cFDR

**Figure 2:**
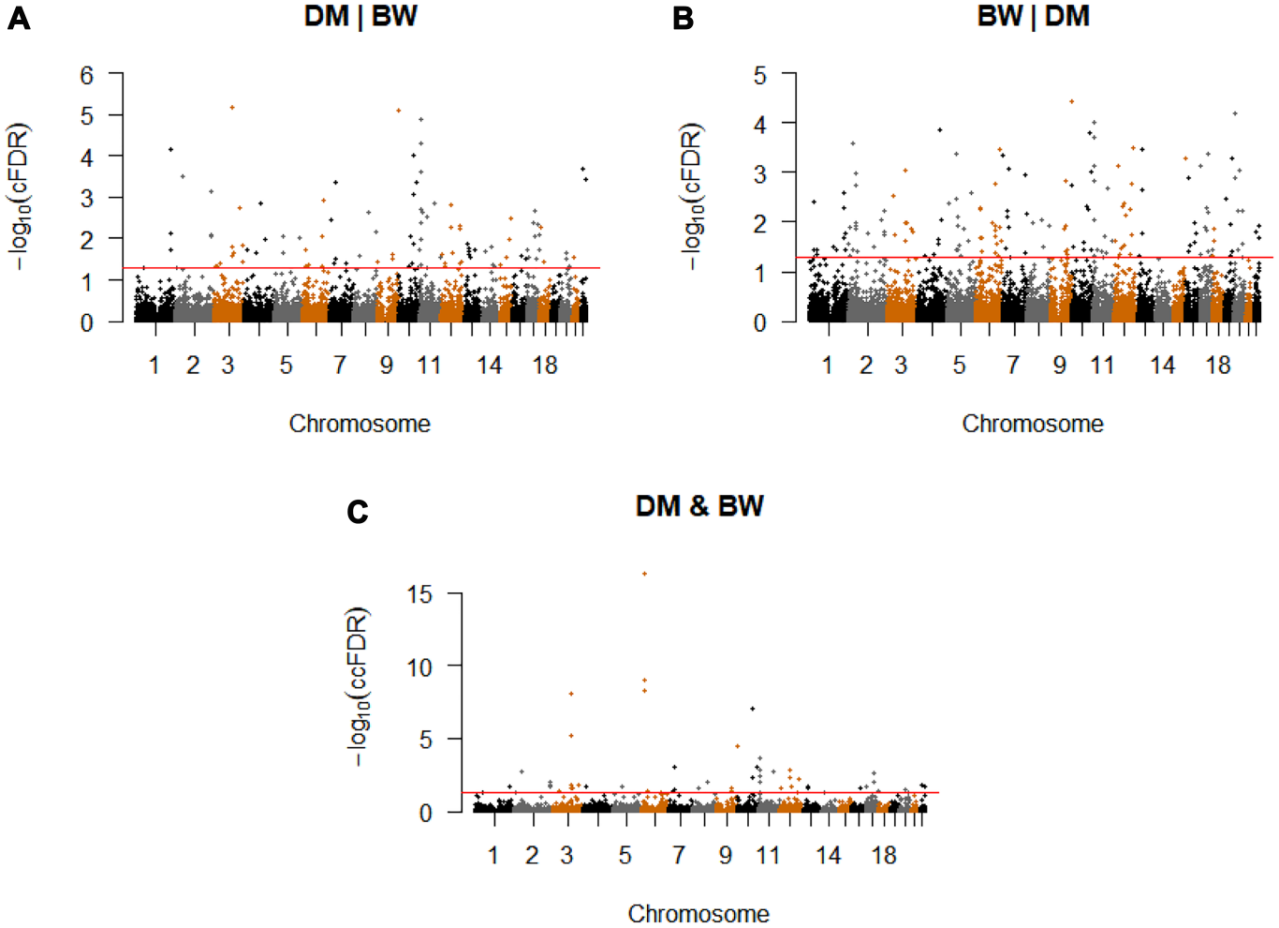
**Conditional Manhattan plot.** Conditional Manhattan plot of conditional -log10(FDR) values for (A) T2D given BW (T2D|BW), (B) BW given T2D (BW|T2D), (C) T2D and BW. The red line marks the conditional -log10(FDR) values of 1.3 corresponds to a cFDR ≤ 0.05.

Conditional on association with BW, we identified 127 significant CpG-SNPs (cFDR ≤ 0.05) for T2D variation, which were located on 21 different chromosomes and annotated to 110 genes (Figure 2A, Supplementary Table 1). Among these significant SNPs, 103 were not reported compared with the original T2D GWAS study [13], our earlier work [12] and other previous studies [15, 27]. Using the more conservative threshold of cFDR ≤ 0.01, 64 significant loci remained.

To explore the potential regulatory functions of these CpG-SNPs, we conducted a series of bioinformatics analyses. Our results found that 27 CpG-SNPs showed significant meQTL effects in whole blood (Supplementary Table 2). Additionally, there were 18 loci that mapped to metaQTLs. Interestingly, two novel SNPs (rs677042 and rs7816345) were associated with bile acids (i.e., ursodeoxycholate, hyodeoxycholate), and a third novel SNP (rs10774563) was related to branched chain fatty acids (i.e., ethylmalonate, methylsuccinate). Bile acid and branched-chain fatty acids are known to be involved in the mechanisms of glucose metabolism [28]. Multiple recent studies reported that short-chain fatty acids and branched short-chain fatty acids may have beneficial health effects on adipocyte lipid and glucose metabolism that can contribute to improved insulin sensitivity in individuals with disturbed metabolism [29, 30]. Furthermore, the SNP rs11659412 (Supplementary Table 3) has associations with methylamine, which has previously been shown to have connections with susceptibility for T2D [31].

We also detected four pathways associated with the metabolites that are linked to the metaQTLs. Pathway analysis suggested that these metabolites were linked mainly to the lipid metabolism (alpha-Linolenic acid metabolism and Glycerophospholipid metabolism), energy metabolism (Methane metabolism), and genetic Information Processing (aminoacyl-tRNA biosynthesis) (Supplementary Table 4). Lastly, we detected the SNPs enriched in the eQTLs, which may regulate gene expression levels. Eight CpG-SNPs showed the eQTL effect and were associated with enhancer, promoter, and DNAse elements in various tissues. Notably, two novel CpG-SNPs, rs7787720, and rs579459 showed meQTL, eQTL, and metaQTL effects simultaneously (Table 2).

**Table 1:** Conjunction cFDR for 55 pleiotropic CpG-SNPs in T2D and BW (ccFDR ≤ 0.05).

**Variant**	**Chr**	**Pos**	**Alt**	**Gene**	**Location**	**eQTL/meQTL/metaQTL**	**SNP Type**	**Gene Type**	**cFDR_T2D**	**cFDR_BW**	**ccFDR**
rs10449766	1	42070125	A/G	HNRNPFP1	28396 upstream	-	novel	novel	4.99E-02	3.73E-02	4.99E-02
rs340883	1	213972363	C/T	PROX1-AS1	non-coding intronic	-	T2D	novel	7.04E-05	2.13E-02	2.13E-02
rs7553890	1	213832562	T/C	PROX1-AS1	non-coding	-	novel	novel	1.89E-02	2.53E-03	1.89E-02
rs1515114	2	226233671	A/G	AC062015.1	48300 downstream	eQ-TL	novel	novel	8.17E-03	1.82E-02	1.82E-02
rs1522812	2	226132738	A/G	AC062015.1	47306 downstream	eQTL	novel	novel	7.43E-04	1.55E-02	1.55E-02
rs2894593	2	226325601	T/C	AC062015.1	140230 upstream	eQTL	novel	novel	9.16E-03	6.05E-03	9.16E-03
rs7605661	2	43397939	T/C	THADA	intronic	meQTL	novel	T2D	3.21E-04	1.87E-03	1.87E-03
rs17361324	3	123412407	C/T	ADCY5	intronic	meQTL	novel	pleiotropic	8.40E-09	1.20E-18	8.40E-09
rs4677887	3	123381376	T/G	ADCY5	intronic	eQTL	pleiotropic	pleiotropic	6.80E-06	2.91E-11	6.80E-06
rs4677889	3	123424425	G/A	ADCY5	intronic	-	novel	pleiotropic	2.53E-02	1.02E-02	2.53E-02
rs569255	3	125207090	G/A	SLC12A8	intronic	eQTL	novel	novel	2.27E-02	1.01E-02	2.27E-02
rs6770420	3	170931960	G/A	KLF7P1	20890 downstream	eQTL,metaQTL	novel	novel	1.75E-03	1.63E-02	1.63E-02
rs6794193	3	47073414	T/C	SETD2	intronic	eQTL,meQTL	novel	novel	3.92E-02	3.02E-03	3.92E-02
rs9289218	3	123345984	C/T	ADCY5	intronic	eQTL	novel	pleiotropic	1.60E-02	8.91E-04	1.60E-02
rs7663887	4	17901297	C/A	LCORL	intronic	eQTL	novel	BW	1.89E-02	5.90E-07	1.89E-02
rs10514870	5	59055501	A/G	PDE4D, AC092343.1	intronic,intronic	-	novel	novel	2.18E-02	4.17E-04	2.18E-02
rs1012635	6	20675064	A/G	CDKAL1	intronic	-	pleiotropic	pleiotropic	2.64E-14	1.00E-09	1.00E-09
rs12526403	6	41676676	C/T	TFEB	7302 downstream	-	novel	novel	4.50E-02	3.83E-02	4.50E-02
rs2206734	6	20694653	C/T	CDKAL1	intronic	meQTL	novel	pleiotropic	9.60E-27	4.80E-17	4.80E-17
rs2745929	6	20754530	T/C	CDKAL1	intronic	-	novel	pleiotropic	2.10E-09	4.77E-09	4.77E-09
rs4897378	6	130217352	C/T	SAMD3	5upstream	eQTL	novel	novel	4.54E-02	1.20E-02	4.54E-02
rs17689040	7	40880714	C/G	SUGCT	19951 upstream	-	novel	novel	3.04E-02	1.31E-02	3.04E-02
rs6948511	7	27939096	T/C	JAZF1	intronic	eQTL, metaQTL	novel	T2D	4.02E-02	5.80E-03	4.02E-02
rs7723	7	44578194	G/A	TMED4	3utr	eQTL, metaQTL	T2D	novel	4.22E-04	8.40E-04	8.40E-04
rs7004862	8	94864735	T/G	INTS8	intronic	eQTL, metaQTL	novel	T2D	2.26E-03	9.35E-03	9.35E-03
rs7816345	8	36988591	C/T	AC090453.1	179 upstream	eQTL, metaQTL	novel	novel	2.68E-02	1.05E-02	2.68E-02
rs10739970	9	94134010	A/G	PTPDC1	24154 upstream	-	novel	novel	3.18E-02	3.78E-02	3.78E-02
rs10990568	9	95651855	A/G	AL354861.2	non-coding intronic	-	novel	novel	2.46E-02	1.19E-02	2.46E-02
rs579459	9	133278724	T/C	ABO	3510 upstream	eQTL,meQTL,metaQTL	novel	T2D	8.22E-06	3.60E-05	3.60E-05
rs2421019	10	122391070	C/T	PLEKHA1	5upstream	eQTL	novel	pleiotropic	1.17E-06	9.45E-04	9.45E-04
rs2488071	10	92739820	A/G	Y_RNA	29212 upstream	eQTL, metaQTL	T2D	novel	1.96E-10	9.00E-08	9.00E-08
rs7070786	10	92363930	C/T	MARCH5	9966 upstream	eQTL	novel	novel	9.54E-05	4.80E-03	4.80E-03
rs1447351	11	92984997	A/G	MTNR1B	3utr	metaQTL	novel	pleiotropic	1.47E-03	2.05E-03	2.05E-03
rs151216	11	2659585	C/T	KCNQ1, KCNQ1OT1	intronic,non-coding	-	novel	pleiotropic	1.33E-05	1.97E-04	1.97E-04
rs163177	11	2817183	T/C	KCNQ1	intronic	eQTL,meQTL	T2D	pleiotropic	2.07E-10	1.52E-03	1.52E-03
rs231354	11	2685121	T/C	KCNQ1, KCNQ1OT1	intronic,non-coding	eQTL,meQTL	pleiotropic	pleiotropic	2.40E-04	9.10E-03	9.10E-03
rs234857	11	2831299	T/C	KCNQ1	intronic	-	T2D	pleiotropic	1.09E-06	4.67E-02	4.67E-02
rs3213225	11	2135306	G/A	IGF2, INS-IGF2	intronic,intronic	eQTL,meQTL	novel	pleiotropic	4.15E-03	9.59E-05	4.15E-03
rs1042725	12	65964567	C/T	HMGA2	3utr,	-	T2D	pleiotropic	1.54E-03	1.90E-29	1.54E-03
rs10774202	12	4168281	A/G	AC007207.1	50149 upstream	-	novel	novel	2.42E-02	2.32E-02	2.42E-02
rs10862960	12	77030355	C/T	E2F7	intronic	eQTL	novel	novel	2.14E-02	7.30E-03	2.14E-02
rs10878353	12	65988752	T/C	HMGA2	22457 upstream	-	novel	pleiotropic	5.34E-03	1.04E-06	5.34E-03
rs4930718	12	123428886	A/G	RILPL2	intronic	eQTL	novel	novel	6.05E-03	3.28E-04	6.05E-03
rs12865243	13	40104683	G/A	LINC00598	non-coding intronic	-	novel	novel	2.44E-02	3.32E-04	2.44E-02
rs9532498	13	40104306	G/C	LINC00598	non-coding intronic	-	novel	novel	2.05E-02	2.22E-03	2.05E-02
rs4625714	16	55607701	C/T	LPCAT2	21031 upstream	-	novel	novel	2.64E-02	1.05E-02	2.64E-02
rs1531798	17	78826049	A/G	USP36	intronic	eQTL	novel	novel	7.84E-03	3.72E-02	3.72E-02
rs198542	17	50567176	G/A	CACNA1G	intronic	-	novel	novel	9.15E-03	6.42E-03	9.15E-03
rs390200	17	7206676	A/G	DLG4	intronic	eQTL	novel	novel	1.88E-02	6.89E-10	1.88E-02
rs6565531	17	81049580	G/A	BAIAP2	intronic	eQTL,meQTL	novel	novel	1.88E-02	3.61E-02	3.61E-02
rs878619	17	50555910	A/G	SPATA20	58 upstream	eQTL,meQTL	novel	novel	2.23E-03	4.24E-04	2.23E-03
rs2426778	20	58718421	G/A	NPEPL1	non-coding	eQTL	novel	novel	4.76E-02	6.10E-03	4.76E-02
rs926345	20	41143307	T/C	PLCG1	intronic	eQTL,meQTL	novel	novel	3.11E-02	9.32E-04	3.11E-02
rs137848	22	50001867	T/C	IL17REL	intronic	eQTL,meQTL	novel	novel	3.55E-04	2.08E-02	2.08E-02
rs6006393	22	30194037	T/C	AC002378.1	non-coding intronic	eQTL	novel	novel	2.14E-04	1.53E-02	1.53E-02

Abbreviations: Chr, chromosome; Pos, chromosomal position (GRCh38/hg38); Alt, reference allele/alter allele; eQTL, expression quantitative trait locus; meQTL, methylation quantitative trait locus (including associated SNPs with an LD *r*^2^ ≥ 0.8, Supplementary Table 3); metaQTL, metabolic quantitative trait locus. T2D, type 2 diabetes; BW, birth weight; cFDR, conditional false discovery rate; ccFDR, conjunctional conditional false discovery rate. The allele was exhibited as reference allele/alter allele; SNP type and gene type means whether identified CpG-SNPs and genes have been reported in previous GWAS or in our previous related cFDR studies.

**Table 2:** Functional annotation for 17 CpG-SNPs showing significant effects in meQTL, eQTL, and metaQTL.

**rsID**	**GENCODE genes**	**Traits**	**meQTL (*p*)**	**eQTL hits**	**mQTL (metabolics)**	**Promoter histone marks**	**Enhancer histone marks**	**DNAse**	**Proteins bound**	**Motifs changed**
rs579459	ABO	Pleiotropic	1.45E-09	5 hits	glycylglycine	BLD	GI	4 tissues	NFYA, POL2	Hmx, Nkx2
rs6446490	PPP2R2C	T2D	2.81E-16	7 hits	-	LIV	4 tissues	4 tissues	7 bound proteins	-
rs7787720	AC005019.2	T2D	2.04E-22	1 hit	salicyluric glucuronide	-	MUS, LNG, SKIN	-	-	Mef2
rs12245680	TCF7L2	T2D	8.59E-05	-	-	15 tissues	17 tissues	29 tissues	FOXA1	-
rs11819995	ETS1	T2D	5.19E-05	-	-	19 tissues	9 tissues	15 tissues	POL2	NRSF
rs2237892	KCNQ1	T2D	1.45E-10	-	#gamma-g, ^**^N2, N2-d	-	5 tissues	KID, MUS	-	8 altered motifs
rs2334499	FAM99B	T2D	4.05E-09	2 hits	-	LIV	ADRL, LIV	MUS, MUS	-	GR, PU.1, RXRA
rs2291725	GIP	T2D	1.62E-06	29 hits	-	11 tissues	14 tissues	22 tissues	16 bound proteins	Sin3Ak-20
rs6687139	LINC01681	BW	1.90E-05	1 hit	-	FAT	7 tissues	4 tissues	-	-
rs863818	PIK3R1	BW	-	-	1-methylxanthine	BLD	15 tissues	HRT	-	-
rs3750640	ASB13	BW	-	2 hits	phenylalanylserine	GI	7 tissues	IPSC, THYM, GI	-	PPAR
rs4980661	AP000439.2	BW	1.02E-04	-	-	SPLN	HRT, MUS, LIV	LNG, BLD	-	-
rs11079803	PNPO	BW	5.25E-09	57 hits	-	19 tissues	13 tissues	11 tissues	-	6 altered motifs
rs4647887	SNHG16	BW	6.23E-20	33 hits	-	5 tissues	16 tissues	14 tissues	GATA1	-
rs2586211	GNAL	BW	-	-	allantoin	LIV	9 tissues	9 tissues	CTCF, CMYC	CEBPB, GATA
rs2261988	UHRF1	BW	1.83E-05	7 hits	-	21 tissues	10 tissues	24 tissues	POL2, POL24H8	4 altered motifs
rs492602	FUT2	BW	2.50E-07	31 hits	^*^ADp	-	-	-	-	Znf143

Abbreviations: meQTL, methylation quantitative trait locus (including associated SNPs with an LD *r*^2^ ≥ 0.8, Supplementary Table 3); eQTL, expression quantitative trait locus; metaQTL, metabolic quantitative trait locus. DNAse, deoxyribonuclease; ^*^ADp, ADpSGEGDFXAEGGGVR; ^#^gamma-g, gamma-glutamylvaline; ^**^N2, N2-d, N2, N2-dimethylguanosine.

### BW loci identified with cFDR

We identified a total of 188 significant CpG-SNPs (cFDR ≤ 0.05) for BW variation on their association with T2D, which were mapped to 20 different chromosomes (Figure 2B, Supplementary Table 5). Other than seven confirmed CpG-SNPs for BW [12, 14], the remaining 182 are novel CpG-SNPs. Using the more conservative threshold of conditional cFDR ≤ 0.01, 85 significant loci remained.

Likewise, we performed the analysis to explore the potential regulatory functions of these identified CpG-SNPs. We found 34 CpG-SNPs that showed significant meQTL effects in whole blood (Supplementary Table 2), and 20 CpG-SNPs that showed metaQTL effects. Among these SNPs identified by the metaQTL analysis, three were associated with bile acids, and one was associated with fatty acids. For the metabolites associated with these CpG-SNPs, two significant pathways were detected, including aminoacyl-tRNA biosynthesis and glycerophospholipid metabolism (Supplementary Table 3, Supplementary Table 4). Lastly, nine novel CpG-SNPs (i.e., rs6687139, rs863818, rs579459, rs3750640, rs4980661, rs11079803, rs4647887, rs2586211, and rs2261988) were identified as eQTL SNPs and were associated with enhancer, promoter, and DNAse elements in various tissues. There was one novel CpG-SNP, rs492602, which showed meQTL, eQTL, and metaQTL effects simultaneously (Table 2). To further verify the eQTL effects of identified loci, we applied LocusCompare method (see methods). Using this method, we identified 11 eQTL loci in T2D and 15 eQTL loci in BW (Supplementary Table 6). Two of these loci are overlapped with our results: *JAZF1* (*p* = 8.7 × 10^−9^), and *PLEKHA1* (*p* = 5.2 × 10^−8^), indicating that these two genes are more likely to be the eQTL loci.

### Pleiotropic loci in T2D and BW identified with ccFDR

We computed ccFDR and constructed a ccFDR Manhattan plot to investigate whether any of the CpG-SNPs were associated with both T2D and BW. We found 55 independent pleiotropic SNPs detected by our analysis (The detailed annotations are listed in Supplementary Table 1 and 5), which were located on 16 chromosomes that reached a significance level of ccFDR ≤ 0.05 (Figure 2C, Table 1). With the more stringent significance threshold of ccFDR ≤ 0.01, 23 pleiotropic CpG-SNPs remained. Among the identified loci, 52 CpG-SNPs were suggested to be novel. In total, five SNPs have been associated with T2D, while none of these SNPs has previously been identified for BW. All the identified CpG-SNPs annotated to 45 different genes, and 35 of these were not detected as pleiotropic genes in previous related research [12]. Finally, we found 35 SNPs have at least one eQTL, meQTL, or metaQTL effect. One pleiotropic CpG-SNP, rs579459 (*ABO*), showed eQTL, meQTL, and metaQTL effects simultaneously (Table 2).

### GO enrichment analysis and protein-protein interaction analysis.

**Figure 3:**
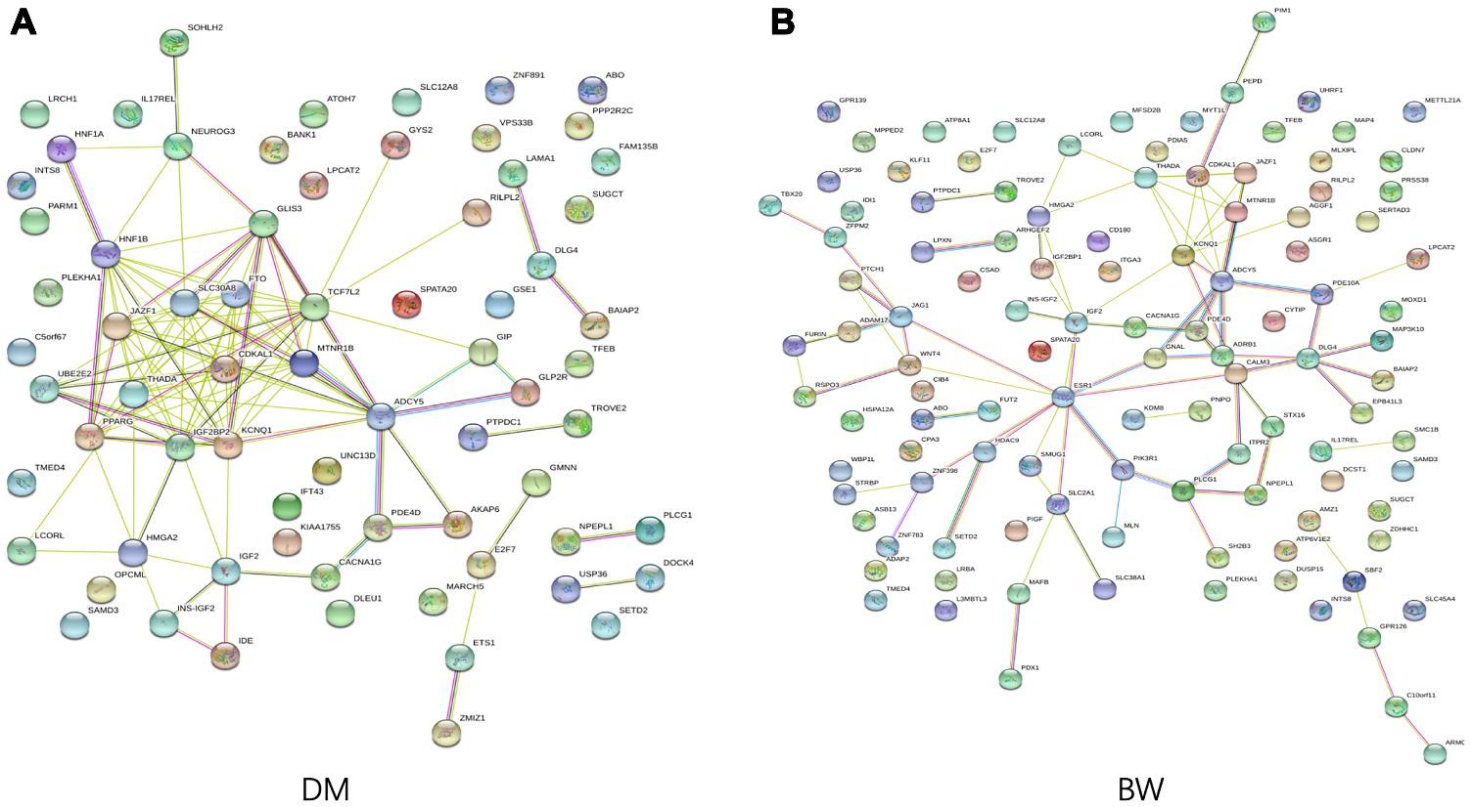
**Functional protein association network analysis.** Connections are based on evidence with a STRING 11.0 summary score above 0.4. Each network nodes represent a gene; edges between nodes indicate protein-protein interactions between protein products of the corresponding genes in (**A**) T2D and (**B**) BW, edge colors indicate the types of interaction.

We conducted GO enrichment analysis for the T2D- and BW- associated genes that were annotated to the identified CpG-SNPs to explore the potential regulatory functions. The identified SNPs were enriched in biological processes related to "response to oxygen-containing compound" and a molecular function of "scaffold protein binding". We also found that genes associated with T2D were significantly enriched in the pathway of "regulation of hormone levels" (FDR = 3.04 × 10^−3^) and "regulation of insulin secretion" (FDR = 4.19 × 10^−3^) (Supplementary Table 7). Using STRING 11.0 database, we performed protein-protein interaction analysis to further investigated the functional partnership among identified T2D- and BW- associated genes (Figure 3**)**, respectively. PPI results showed several genes were well-connected in the interaction network in both traits, such as *ADCY5*, *KCNQ1*, *IGF2*, and *CDKAL1*, suggesting these genes are essential in the genetic network that coupling of both traits.

### Results of the validation study

For cFDR analysis between BW and FG, we observed similar significant separation between the different curves, which indicates a strong pleiotropy between those two traits (Supplementary Figure 1A and 1B). Conditional on association with FG, we identified 160 significant CpG-SNPs (cFDR ≤ 0.05) for BW variation, and 131 of them were replicated compared with the main cFDR analysis. We identified a total of 104 significant CpG-SNPs for FG variation on their association with BW, and 25 of them were replicated. And we replicated 18 pleiotropic CpG-SNPs for FG and BW (Supplementary Table 8).

For cFDR analysis between BW and FI, we identified clearly separation between the different curves, which indicates a strong pleiotropy between those two traits (Supplementary Figure 1C and 1D). Conditional on association with FI, we identified 140 significant CpG-SNPs for BW variation, and 125 of them were replicated compared with the main cFDR analysis. We identified a total of 13 significant CpG-SNPs for FI variation on their association with BW, and 5 of them were replicated. And we replicated 3 pleiotropic CpG-SNPs for FI and BW (Supplementary Table 9).

For cFDR analysis between BW_maternal and T2D_corBMI, significant deflection between curves suggested strong pleiotropy between those two phenotypes (Supplementary Figure 2A and 2B). Conditional on association with T2D_corBMI, we identified 90 CpG-SNPs for BW_ maternal variation, and 13 of them were replicated compared with the main cFDR analysis. We identified a total of 622 significant CpG-SNPs for T2D_corBMI variation on their association with BW_ maternal, and 79 of them were replicated. And we replicated 8 pleiotropic CpG-SNPs for BW_ maternal and T2D_corBMI (Supplementary Table 10).

For cFDR analysis between BW_fetal and T2D_corBMI, similar deflection between curves demonstrated significant pleiotropy between those two phenotypes (Supplementary Figure 2C and 2D). Conditional on association with T2D_corBMI, we identified 133 CpG-SNPs for BW_fetal variation, and 59 of them were replicated compared with the main cFDR analysis. We identified a total of 669 significant CpG-SNPs for T2D_corBMI variation on their association with BW_ fetal, and 87 of them were replicated. And we replicated 25 pleiotropic CpG-SNPs for BW_ fetal and T2D_corBMI (Supplementary Table 11).

### MR results

Finally, 46 independent SNPs were left for MR analysis (Table 3). IVW results suggested causal association between BW and T2D (OR = 1.554, se = 0.207, 95% CI (1.036, 2.330), *P* = 0.033), MR-Egger regression demonstrated no pleiotropy among selected IVs (*P* = 0.450). However, other approaches did not identify any causal association (Table 4). Our bi-directional MR analysis also suggested no causal association between T2D and BW.

**Table 3:** Characteristics of the instrumental variables used in MR analysis.

**SNP**	**ea_E**	**oa_E**	**ea_O**	**oa_O**	**beta.E**	**beta.O**	**eaf.E**	**eaf.O**	**se.O**	**pval.O**	**outcome**	**se.E**	**pval.E**	**exposure**
rs10818797	C	T	C	T	0.0345	0.0953102	0.14	0.141	0.076	0.3	T2D	0.0054	1.20E-10	BW
rs10830963	G	C	G	C	0.0232	0	0.28	0.276	0.039	0.27	T2D	0.0042	2.90E-08	BW
rs10872678	C	T	C	T	-0.0375	-0.040822	0.28	0.277	0.04	0.31	T2D	0.0041	6.90E-20	BW
rs10935733	C	T	C	T	-0.0221	-0.0953102	0.59	0.606	0.034	0.092001	T2D	0.0039	9.20E-09	BW
rs111778406	G	A	G	A	0.0492	-0.0202027	0.068	0.072	0.13	0.89	T2D	0.0075	5.80E-11	BW
rs113086489	T	C	T	C	0.0307	0	0.56	0.545	0.034	0.94	T2D	0.0038	9.10E-16	BW
rs11720108	T	C	T	C	0.046	0	0.23	0.249	0.045	0.61	T2D	0.0043	3.40E-26	BW
rs11765649	C	T	C	T	-0.0267	0	0.25	0.263	0.042	0.37	T2D	0.0043	5.80E-10	BW
rs1187118	T	A	T	A	-0.0299	0	0.83	0.833	0.058	0.73	T2D	0.0051	3.60E-09	BW
rs12543725	A	G	A	G	-0.0231	0	0.41	0.412	0.033	0.41	T2D	0.0038	1.20E-09	BW
rs12906125	A	G	A	G	-0.0228	-0.0100503	0.32	0.326	0.037	0.88	T2D	0.004	1.70E-08	BW
rs13266210	G	A	G	A	-0.0308	-0.0512933	0.21	0.212	0.051	0.36	T2D	0.0045	1.30E-11	BW
rs134594	T	C	T	C	-0.0227	0.0100503	0.65	0.65	0.036	0.77	T2D	0.004	1.00E-08	BW
rs1351394	C	T	C	T	-0.0436	0	0.51	0.511	0.032	0.760001	T2D	0.0037	1.90E-32	BW
rs1411424	A	G	A	G	0.0212	0.0202027	0.52	0.524	0.033	0.62	T2D	0.0038	2.20E-08	BW
rs1415701	A	G	A	G	-0.0253	0	0.26	0.269	0.041	0.9	T2D	0.0043	2.60E-09	BW
rs144843919	A	G	A	G	-0.066	-0.0100503	0.035	0.035	0.27	0.98	T2D	0.0116	1.40E-08	BW
rs17034876	T	C	T	C	0.0471	0.0304592	0.7	0.699	0.039	0.42	T2D	0.0042	2.60E-29	BW
rs1819436	C	T	C	T	0.0329	0.0100503	0.87	0.877	0.076	0.93	T2D	0.0057	6.30E-09	BW
rs2131354	A	G	A	G	0.0259	0	0.53	0.526	0.033	0.52	T2D	0.0037	4.10E-12	BW
rs2168443	A	T	A	T	-0.0228	0.0725707	0.62	0.621	0.035	0.053001	T2D	0.0039	3.50E-09	BW
rs2229742	C	G	C	G	-0.036	-0.040822	0.13	0.104	0.084	0.62	T2D	0.006	2.20E-09	BW
rs2306547	T	C	T	C	-0.0211	0	0.46	0.467	0.033	0.92	T2D	0.0037	1.80E-08	BW
rs2473248	C	T	C	T	0.0325	0	0.87	0.881	0.079	0.94	T2D	0.0057	1.00E-08	BW
rs2497304	T	C	T	C	-0.0282	0	0.48	0.478	0.033	0.97	T2D	0.0037	2.60E-14	BW
rs28530618	G	A	G	A	-0.0261	-0.0953102	0.51	0.529	0.033	0.041	T2D	0.0038	7.70E-12	BW
rs2946179	C	T	C	T	0.024	0	0.73	0.74	0.042	0.27	T2D	0.0042	1.30E-08	BW
rs35261542	A	C	A	C	-0.0444	-0.127833	0.27	0.263	0.039	0.00063	T2D	0.0041	4.40E-27	BW
rs3753639	C	T	C	T	0.0306	0.0953102	0.24	0.245	0.046	0.25	T2D	0.0045	7.30E-12	BW
rs3780573	A	G	A	G	0.0555	0.0953102	0.096	0.096	0.099	0.22	T2D	0.0064	7.00E-18	BW
rs4144829	T	C	T	C	-0.0341	0	0.73	0.739	0.043	0.56	T2D	0.0042	5.30E-16	BW
rs6016377	T	C	T	C	0.0239	0	0.43	0.446	0.035	0.53	T2D	0.0039	9.50E-10	BW
rs6040076	C	G	C	G	0.0231	-0.0512933	0.49	0.494	0.033	0.1	T2D	0.0039	2.00E-09	BW
rs72480273	C	A	C	A	0.0313	-0.0512933	0.17	0.189	0.056	0.38	T2D	0.0051	8.00E-10	BW
rs72833480	A	G	A	G	0.0226	-0.0304592	0.29	0.295	0.039	0.49	T2D	0.0041	4.60E-08	BW
rs72851023	T	C	T	C	0.0476	0	0.073	0.077	0.13	0.74	T2D	0.0075	2.90E-10	BW
rs7402982	G	A	G	A	-0.0232	0.0100503	0.57	0.586	0.034	0.67	T2D	0.0039	2.30E-09	BW
rs740746	A	G	A	G	0.0364	0	0.73	0.734	0.041	0.62	T2D	0.0042	3.80E-18	BW
rs753381	C	T	C	T	-0.0205	0	0.55	0.55	0.033	0.52	T2D	0.0037	2.80E-08	BW
rs7575873	G	A	G	A	-0.0384	-0.0618754	0.12	0.13	0.071	0.42	T2D	0.0057	1.20E-11	BW
rs7854962	G	C	G	C	-0.0279	-0.040822	0.22	0.216	0.047	0.36	T2D	0.0046	1.90E-09	BW
rs79237883	C	T	C	T	0.0371	-0.0833816	0.08	0.076	0.1	0.39	T2D	0.0067	3.50E-08	BW
rs7964361	A	G	A	G	0.0391	-0.105361	0.085	0.088	0.096	0.25	T2D	0.0067	4.70E-09	BW
rs798498	G	T	G	T	-0.0229	0	0.31	0.307	0.038	0.709999	T2D	0.004	1.30E-08	BW
rs7998537	A	G	A	G	-0.0222	0	0.32	0.321	0.037	0.35	T2D	0.004	3.90E-08	BW
rs854037	G	A	G	A	-0.0268	0.0953102	0.19	0.186	0.055	0.37	T2D	0.0048	2.20E-08	BW
rs900399	G	A	G	A	-0.0523	0	0.39	0.393	0.038	0.47	T2D	0.0039	2.20E-41	BW
rs9368777	C	G	C	G	0.0215	0.0304592	0.58	0.575	0.033	0.31	T2D	0.0038	2.20E-08	BW

Notes:

E: exposure; O: outcome; ea: effect_allele; oa: other_allele.

**Table 4:** MR analysis results.

**Outcome**	**Exposure**	**Method**	**nsnp**	**b**	**se**	**pval**	**or**	**or_lci95, or_uci95**
T2D	BW	MR Egger	46	0.968	0.723	0.187	2.632	0.639, 10.845
T2D	BW	Weighted median	46	0.000	0.309	1.000	1.000	0.546, 1.831
T2D	BW	Inverse variance weighted	46	0.441	0.207	0.033	1.554	1.036, 2.330
T2D	BW	Simple mode	46	0.009	0.527	0.986	1.009	0.359, 2.834
T2D	BW	Weighted mode	46	0.009	0.431	0.983	1.009	0.434, 2.347

## DISCUSSION

In this study, by performing the cFDR on the two independent GWAS datasets from T2D and BW, we identified 103 novel loci for T2D and 182 for BW when focusing on CpG-SNPs. Meanwhile, we identified 55 pleiotropic CpG-SNPs suggesting a shared genetic mechanism among them. Interestingly, only three of these CpG SNPs were identified as pleiotropic loci in the previous studies.

Since the genetic variants located at CpG-SNPs could affect the gene expression and regulation via epigenetic mechanisms, we investigated these 103 CpG-SNPs, which were regarded as novel SNPs associated with T2D. Among those CpG-SNPs, 55 showed at least one effect on meQTL, metaQTL, and/or eQTL, and 35 of them were located at novel risk genes for T2D. For example, rs11073964 is a novel CpG-SNP which showed both eQTL and meQTL effects and mapped to *VPS33B* (15q26.1). The relationship between *VPS33B* and T2D is unknown, although *VPS33B* is expressed in pancreas tissue, and encodes Vascular Protein Sorting-associated protein 33B [32]. The function of *VPS33B* refers to intracellular protein trafficking and membrane fusion mechanisms [33]. It also plays critical roles in bile acids metabolism [34], which could contribute to the regulation of glucose homeostasis [28]. Therefore, it is conceivable that *VPS33B* might influence pancreatic cell function through epigenetic mechanisms.

Another novel CpG-SNP (rs12786533), also showed both meQTL and eQTL effects and was located at the gene *KCNQ1DN* (*KCNQ1* downstream neighbor). *KCNQ1DN*, which imprinted and mapped between *CDKN1C* and *KCNQ1* on chromosome 11p15.5, is usually associated with Wilms' tumor [35]. Other imprinted genes in 11p15.5, including *KCNQ1* and *IGF2*, are candidates for involvement in T2D [36, 37]. We also detected seven CpG-SNPs (rs151216, rs163171, rs163177, rs2237892, rs231354, rs234857, and rs3852527) which were annotated to *KCNQ1*. Two of these loci (rs163177 and rs231354) were shown to have eQTL and meQTL effects, and another SNP (rs2237892) was shown to have metaQTL effect. It is plausible that these multiple neighboring CpG-SNPs might synergistically regulate gene expression and play some roles in T2D.

We also investigated the significant CpG-SNPs associated with BW conditioned on T2D, in which 120 CpG-SNPs were novel loci and annotated to genes that were not reported in previous study [13]. All these SNPs showed at least one effect on eQTL, meQTL, and/or metaQTL. There were two notable SNPs, rs3184504, which was located on the gene *SH2B3*, and rs492602, which was located on the gene *FUT2*. Both SNPs showed eQTL, meQTL, and metaQTL effects simultaneously. The gene *SH2B3* acts as a negative regulator of cytokine signaling and cell proliferation [38], which is known to be associated with type 1 diabetes and celiac disease [39]. The gene *FUT2* encodes a specific fucosyltransferase enzyme, which is crucial for the synthesis of histo-blood group antigens [40]. Whether these genes have a function on BW is unclear, but our findings imply that epigenetic alteration deserves attention and presents new insights for further exploration.

Importantly, 19 of the 55 pleiotropic variants were novel loci that showed at least one QTL effect. Notably, seven of these 19 loci (rs6770420 in *KLF7P1*, rs6794193 in *SETD2*, rs7816345 in *AC090453.1*, rs6565531 in *BAIAP2*, rs878619 in *SPATA20,* rs926345 in *PLCG1*, and rs137848 in *IL17REL*) showed eQTL and either metaQTL or meQTL effect. These facts suggest that these SNPs might be involved in the shared pathogenesis of both T2D and BW.

There are several advantages in the current study. First, identifying shared genetic factors between these two traits can facilitate our understanding of the genetic correlation between BW and T2D. To our knowledge, this study is the first to use a targeted cFDR method by focusing on functional CpG-SNPs that are associated with both T2D and BW. Compared with our previous work on the same two traits [12], this work was based on the updated GWAS datasets with a larger population and sophisticated study design [13, 14]. Second, in this study, we only focused on functional genetic variants-CpG-SNPs, which not only significantly reduced the multiple testing burden but also shed light on the biological interpretation of the results. Furthermore, we performed the analysis on meQTL, metaQTL, and eQTL effects, which facilitate the identification of candidate functional variants associated with T2D and/or BW. Third, our MR analysis IVW approach showed causal association between BW and T2D while other methods did not, therefore, we cannot define the direct causal relationship between BW and T2D. This result suggested that instead of acting like a direct risk factor, the BW might regulate T2D through multiple intermediate variants such as DNA methylation or regulation of metabolism, as we demonstrated in this study. Fourth, considering BW is influenced both by inherited fetus genotypes and maternal genotypes, we performed validation analysis by using GWAS datasets of direct fetal and indirect maternal genetic effects, which further support the story. Finally, multiple validations in different GWAS datasets were performed in the current study to partially support our findings, which demonstrated the credibility and significance of these findings.

There are also some limitations to our study. First, we are unable to evaluate the effect estimates of pleiotropic SNPs on the traits due to our inability to access the individual-level data. Second, it is difficult to distinguish between the pleiotropic scenarios where a SNP directly influences both BW and T2D, or the SNP affects BW, and the resulting change in phenotype influences T2D susceptibility. However, our MR analysis results may suggest that the SNPs affect T2D susceptibility via affecting BW. Thirdly, although using LD *r*^2^ ≥ 0.2 as the threshold for SNP pairwise pruning is a common practice in similar integration studies [41–43], some of our findings could still be secondary to the signals, especially for SNPs identified in the same gene without special features such as eQTL (e.g., rs151216, rs234857 in *KCNQ1*). However, since the identified SNPs are functional CpG-loci, the CpG-SNPs identified in this study are more likely to be the leading signals, compared with the previous results. Finally, these findings are based on a bioinformatics analysis of GWAS data. Without further molecular validation, some of these results are suggestive rather than conclusive, such as suggested CpG-loci or functional genes. The aim of our study was to find more potential novel T2D associated variants, so we hope that this limitation could partially be addressed in the future by follow up with fine-mapping studies or molecular validation experiments.

In conclusion, by using cFDR method on functional CpG-SNPs, we successfully improved the identification of novel genetic variants of both T2D and BW. Our findings offer an improved understanding of the potential shared genetic mechanisms in T2D and BW, which may provide a new direction for further biological studies and clinical trials.

## MATERIALS AND METHODS

### GWAS datasets

We obtained GWAS summary statistics for T2D and BW from publicly available sources [13, 14]. The dataset for T2D contained meta-analysis summary statistics from 18 studies performed by DIAbetes Genetics Replication and Meta-analysis (DIAGRAM) Consortium (*n* = 26,676 case and 132,532 control, European) [13]. The dataset for BW contained meta-analysis summary statistics from 37 studies conducted by Early Growth Genetics (EGG) Consortium (*n* = 153,781, predominantly European) [14]. Both of these datasets have large enough sample sizes (*n* > 100,000) for statistical power. Each dataset contains summary statistics for each SNP with the *p* values that have undergone genomic control at the individual study level. The detailed inclusion criteria and phenotype characteristics from different GWAS are described in the original publications.

### Identification of potentially functional CpG-SNPs

The CpG-SNPs in the human genome were identified by interrogating the comprehensive catalog of both common and rare genetic variants from the 1000 Genomes reference panel [44], and our in-house whole-genome high-coverage deep re-sequencing study [45]. A SNP is defined as a CpG-SNP if it introduces or disrupts a CpG site. A total of 50,278,228 CpG-SNPs was identified throughout the human genome. The details of the identification of potentially functional CpG-SNPs were described in our previous study [46].

### Data processing

First, we combined the 8,099,761 common SNPs included in these two datasets, then overlapped these common SNPs with the above-identified CpG-SNPs and retrieved a total of 2,478,365 common CpG-SNPs with association summary statistics for both T2D and BW. We then used HapMap 3 genotypes as a reference, and performed a linkage disequilibrium (LD) based pruning method by PLINK 1.9 to remove pairs of SNPs with substantial correlations [47]. The process begins using a window of 50 SNPs, where LD between each pair of SNPs is calculated. If pairs have an R^2^ > 0.2, one of that pair of SNPs is removed. Following this initial removal of SNPs, the window shifts 5 SNPs forward, and the process is repeated until there are no pairs of SNPs that are in high LD. After pruning, 96,312 independent CpG-SNPs remained to be used in the subsequent analysis.

### Statistical analysis

We constructed conditional quantile-quantile plots (Q-Q plot) to evaluate the enrichment of pleiotropic effects by evaluating the increase in the number of trait-associated SNPs for the first trait (principal trait) when conditioning on SNPs with varying strengths of association in the second trait (conditional trait). We also constructed fold-enrichment plots, which quantify the pleiotropic enrichment within each conditional subset compared with the baseline group, which includes all SNPs.

By leveraging two GWASs from T2D and BW, we applied the cFDR approach to obtain the probability that a random SNP is null for association with the principal phenotype given that the *p*-values for the principal and conditional phenotypes are both less than observed *p*-values. The method was applied for both orderings of the two phenotypes, cFDR(T2D|BW) and cFDR(BW|T2D). Then, we computed the conjunction cFDR (ccFDR), taken to be the maximum of the two cFDR values, to identify pleiotropic SNPs for both T2D and BW. Finally, we present conditional Manhattan plots to visualize the localization of the SNPs associated with T2D conditional on the strength of association with BW and the reverse. We also present a conjunction Manhattan plot to visualize the locations of the variants with a pleiotropic effect on both phenotypes. We identified a SNP as novel if it has not been reported in previous GWASs [13, 14] or our previous cFDR studies [12]. The details were presented in the Supplementary Materials and Methods.

### Functional annotation of the pleiotropic CpG-SNPs

To explore the biological functions of the individual trait associated CpG-SNPs and pleiotropic CpG-SNPs, we annotated each identified CPG-SNP to corresponding DNA features or regulatory elements using functional analysis tools such as HaploReg (http://www.broadinstitute.org/mammals/haploreg/haploreg.php) and SNPnexus (http://www.snp-nexus.org/). These tools provide the ENCODE [48] and RoadMap [49] annotations for the CpG-SNPs of interest as well as other SNPs in high LD (*r*^2^ ≥ 0.8).

We further determined whether the identified CpG-SNPs or other SNPs in high LD (*r*^2^ ≥ 0.8) have expression quantitative trait loci (eQTL), methylation QTL (meQTL), or metabolic QTL (metaQTL) effects. First, we obtained the eQTL hits from HaploReg based on GTEx and other eQTL results. Then, we acquired the independent cis- and trans- meQTLs in whole blood from Bonder's study (https://genenetwork.nl/biosqtlbrowser/). Last, we obtained the metaQTLs from SNiPA (https://snipa.helmholtz-muenchen.de/snipa3/), which summarized recently published metaQTL studies. We also used the metabolites associated with these metaQTLs to perform a pathway analysis using MetaboAnalyst 4.0 (https://www.metaboanalyst.ca/). The eQTL results were further confirmed using a web tool "LocusCompare" [50]. This method calculates the colocalization between GWAS and eQTL results and identifies significant loci (GWAS lead SNP p-value < 5 × 10^−8^ and eQTL lead SNP *p*-value < 1 × 10^−6^). We used the original GWAS summary statistics we chose in this study as GWAS input, while using eQTL from adipose tissue as eQTL input (eQTL_ Adipose_Subcutaneous_GTEx_v7) for BW and T2D are closely associated with adipocyte biology [51, 52].

The gene ontology (GO) terms database (http://omicslab.genetics.ac.cn/GOEAST/index.php) was used to perform gene enrichment analysis among the list of genes associated with pleiotropic CpG-SNPs. Among the significant genes we identified gene sets enriched in certain biological processes, cellular components, and molecular functions. To investigate the interaction and functional relationship of the identified T2D and BW genes, protein-protein interaction analyses were constructed by using the STRING 11.0 database (http://string-db.org/).

### Validation study using different GWAS datasets

Considering the long duration of action between BW and T2D, we re-performed cFDR analysis between BW and diabetes-related indicators (fasting glucose (FG), and fasting insulin (FI) [53]) to validate the results of the main cFDR analysis, and QQ plots were also generated to demonstrate the pleiotropic enrichment.

Additionally, considering BMI may be highly related with both birth weight and T2D, and BW is influenced both by inherited fetus genotypes and maternal genotypes with intrauterine environment. We further conducted cFDR analysis between BW_maternal [54] and BW_fetal [54] with T2D with correction of BMI (T2D_corBMI) [55], separately.”

### Bi-directional Mendelian Randomization (MR) analysis

First, we selected independent SNPs (*r*^2^ < 0.001) that achieved genome-wide significance (*p* < 5 × 10−8) in the BW GWAS datasets as instrumental variables (IVs), then summary statistics of those SNPs were further extracted from T2D GWAS datasets. Next, inverse variance weighted (IVW) regression [56] was performed as main MR analysis to estimate the causal relationship between BW and T2D. MR-Egger regression [57] was performed to estimate the pleiotropy effect among selected SNPs. Simple mode, weighted mode and weighted median [56] were then conducted to validate the main results. *P* values less than 0.05 was considered significant. Then bi-directional MR analysis was repeated using T2D as exposure, BW as outcome.

### Ethics approval and patient consent

We obtained genome-wide association study (GWAS) results published online. The relevant institutional review boards or ethics committees approved the research protocol of the individual GWAS used in the current analysis, and all human participants gave written informed consent, which was demonstrated in the respective original papers.

## Supplementary Materials

Supplementary Materials and Methods

Supplementary Figures

Supplementary Tables
