# Supplementary material for: Identification of novel functional CpG-SNPs associated with Type 2 diabetes and birth weight
Source: Aging (Albany NY). 2021 Apr 4;13(7):10619–58. doi: 10.18632/aging.202828 (PMC8064204; doi:10.18632/aging.202828)
Supplement: Supplementary Materials and Methods [file aging-13-202828-s001.pdf]

SUPPLEMENTARY MATERIALS AND METHODS

Pleiotropic enrichment estimation

We performed conditional Q-Q plots based on varying levels of significance in the conditional phenotype to visualize the difference between observed distribution in the principal trait and theoretical distribution. We plotted the QQ curve for the quantile of nominal -log10(*p*) values for the association of the subset of SNPs that were below each different significance threshold in the conditional phenotype. The quantile of the nominal *p*-values are plotted on the x-axis, and the nominal -log10(*p*) values are plotted on the y-axis for T2D and BW respectively. Under the null hypothesis, the strength of pleiotropy enrichment can be reflected by the degree of the leftward shift from the null line. The Q-Q plot falls on the X = Y, which means no enrichment of pleiotropic genetic effect. By contrast, an earlier leftward shift from the null line indicates the existence of pleiotropic enrichment. Greater spacing in the Q-Q plots shows a stronger trend of pleiotropic enrichment shared between the principal and conditional phenotypes.

Then, we presented fold-enrichment plots with “ggplot2 package in R software” [1] to assess further the pleiotropic enrichment between T2D and BW. The plots were formed by nominal -log10(*p*) values at different stratifications which were divided by the *p*-value of SNPs for the conditional phenotype ($p \le 1$; $p \le 0.1$; $p \le 0.01$; $p \le 0.001$). Nominal *p* values (-log10(*p*)) are plotted on the x-axis and fold enrichments are plotted on the y-axis. In each cut-off category, we computed the fold-enrichment values (En, as defined below) for all possible *p* values on the x-axis (between 0 and 10),

$$\mathrm{En[i]} = \frac{N_i}{N_o}$$

and N_i is the proportion of SNPs with -log10(*p*) $\ge x$, N_0 is the number of all SNPs in each cut-off category, and *i* is from 1 to N_0. We can observe an upward shift from the expected baseline as the presence of pleiotropy. Also, the greater separation between different stratification indicated a stronger pleiotropy.

The calculation of cFDR and conjunctional cFDR (ccFDR)

The cFDR is an extension of traditional FDR, and this method is well-established and has been widely applied [2], [3–5]. We performed to integrating the two independent GWASs with summary statistics to assess the probability that an SNP has a false positive association with the principal phenotypes under the given *p*-value for both the principal and conditional phenotypes are smaller than the pre-defined significance thresholds. cFDR was expressed as:

$$\mathrm{cFDR}(p_i|p_j) = \Pr\,(H_0^{(i)}|P_i \le p_i, P_j \le p_j)$$

Where p_i is the strength of association for the SNP with the ‘principal phenotype’, and p_j is the strength of association for the same SNP with the ‘conditional phenotype’. Then the $H_0^{(i)}$ stand for the null hypothesis that there is no association between this given SNP and the principal trait. After the data preparation, we computed the cFDR for each SNP where T2D is the principal phenotype conditioned on the strength of association with BW (T2D|BW) and vice versa (BW|T2D). Using this approach, we identified the loci significantly associated with T2D and BW (FDR < 0.5), respectively.

After the calculation of cFDR, we computed the conjunction cFDR (ccFDR) to find the pleiotropic loci. The maximum cFDR value of the two traits was token as the ccFDR value of each variant. An SNP with the ccFDR value smaller than 0.05 was considered to be significantly associated with both T2D and BW.

Manhattan plots for conditional statistics and conjunction statistics

Using “qqman” package in R software [6], we constructed Manhattan plots to visualize the locations of the genetic markers. All SNPs were present in relation to their chromosomal locations. We plotted locations of the 22 chromosomal on the x-axis, and plotted the −log10 the SNPs' values on the y-axis. The SNP with a -log10(cFDR) ≥ 1.3 was considered as a locus associated with the principal phenotype given the conditional phenotype. Then, an SNP with − log10 conjunction FDR value is > 1.3 was determined to be associated with both the principal trait.

SUPPLEMENTARY REFERENCES

1. Lin X, Peng C, Greenbaum J, Li ZF, Wu KH, Ao ZX, Zhang T, Shen J, Deng HW. Identifying potentially common genes between dyslipidemia and osteoporosis using novel analytical approaches. Mol Genet Genomics. 2018; 293:711–23. https://doi.org/10.1007/s00438-017-1414-1 PMID:29327327

2. Andreassen OA, Thompson WK, Schork AJ, Ripke S, Mattingsdal M, Kelsoe JR, Kendler KS, O'Donovan MC, Rujescu D, Werge T, Sklar P, Roddey JC, Chen CH, et al.

Improved detection of common variants associated with schizophrenia and bipolar disorder using pleiotropy-informed conditional false discovery rate. PLoS Genet. 2013; 9:e1003455. https://doi.org/10.1371/journal.pgen.1003455 PMID:23637625

3. Greenbaum J, Wu K, Zhang L, Shen H, Zhang J, Deng HW. Increased detection of genetic loci associated with risk predictors of osteoporotic fracture using a pleiotropic cFDR method. Bone. 2017; 99:62–68. https://doi.org/10.1016/j.bone.2017.03.052 PMID:28373146

4. Zeng CP, Chen YC, Lin X, Greenbaum J, Chen YP, Peng C, Wang XF, Zhou R, Deng WM, Shen J, Deng HW. Increased identification of novel variants in type 2 diabetes, birth weight and their pleiotropic loci. J Diabetes. 2017; 9:898–907. https://doi.org/10.1111/1753-0407.12510 PMID:27896934

5. Zhang Q, Liu HM, Lv WQ, He JY, Xia X, Zhang WD, Deng HW, Sun CQ. Additional common variants associated with type 2 diabetes and coronary artery disease detected using a pleiotropic cFDR method. J Diabetes Complications. 2018; 32:1105–112. https://doi.org/10.1016/j.jdiacomp.2018.09.003 PMID:30270018

6. Turner SD. qqman: an R package for visualizing GWAS results using QQ and manhattan plots. Biorxiv. 2014:005165. https://doi.org/10.1101/005165
