# Supplementary material for: Identification of novel functional CpG-SNPs associated with Type 2 diabetes and birth weight
Source: Aging (Albany NY). 2021 Apr 4;13(7):10619–58. doi: 10.18632/aging.202828 (PMC8064204; doi:10.18632/aging.202828)
Supplement: Supplementary Figures [file aging-13-202828-s002.pdf]

SUPPLEMENTARY FIGURES

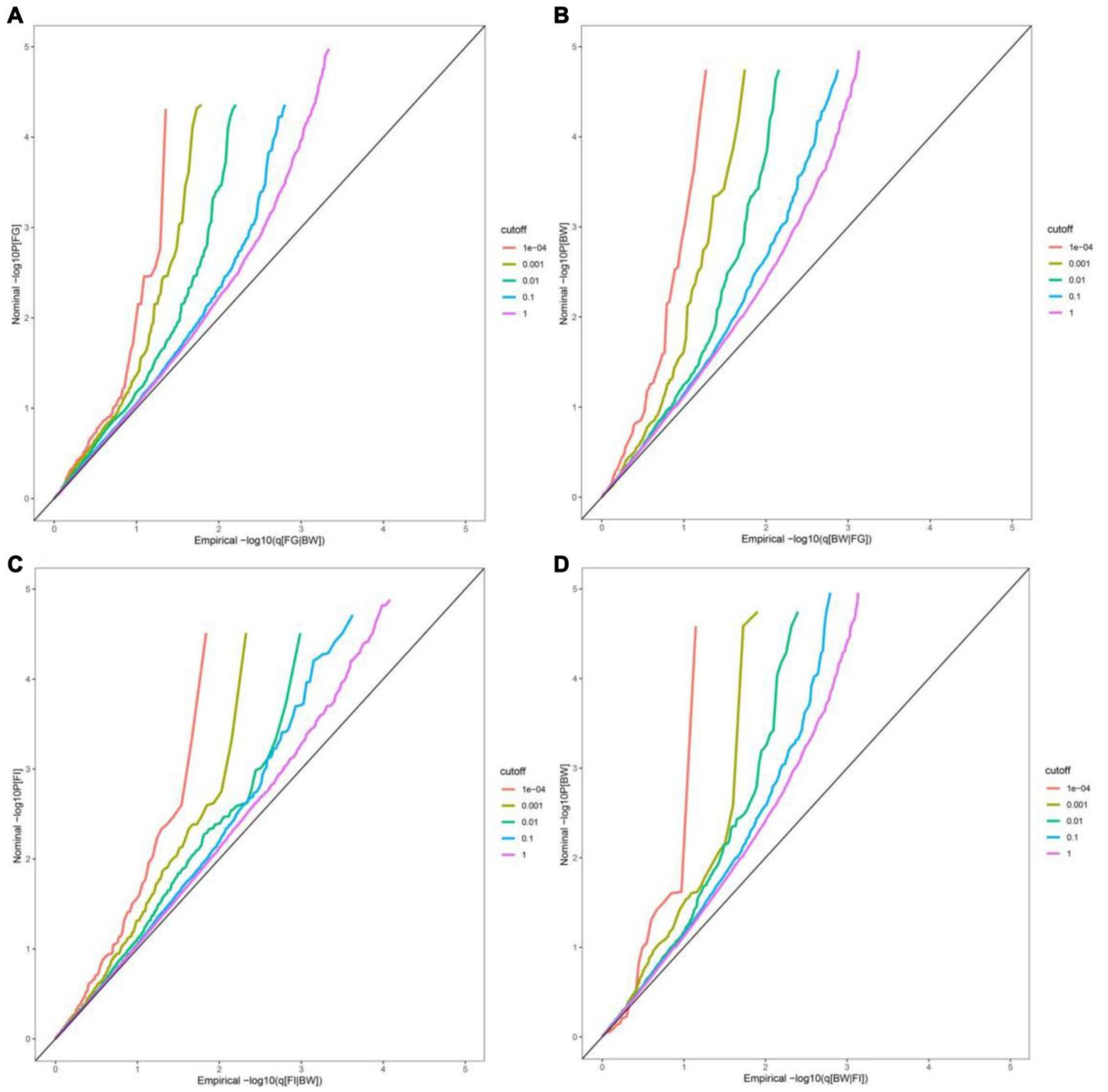

Supplementary Figure 1. Stratified Q-Q plots. Stratified Q-Q plots of nominal versus empirical -log10(*p*) values in FG (**A**) as a function of the significance of the association with BW at the level of -log10(*p*) > 0, -log10(*p*) > 1, -log10(*p*) > 2, -log10(*p*) > 3 corresponding to $p \leq 1$, $p \leq 0.1$, $p \leq 0.01$, $p \leq 0.001$, and $p \leq 0.0001$, respectively. and (**B**) reversely BW as a function of the significance of the association with FG. in (**C**) FI as a function of significance of the association with BW; and in (**D**) BW as a function of significance with FI.

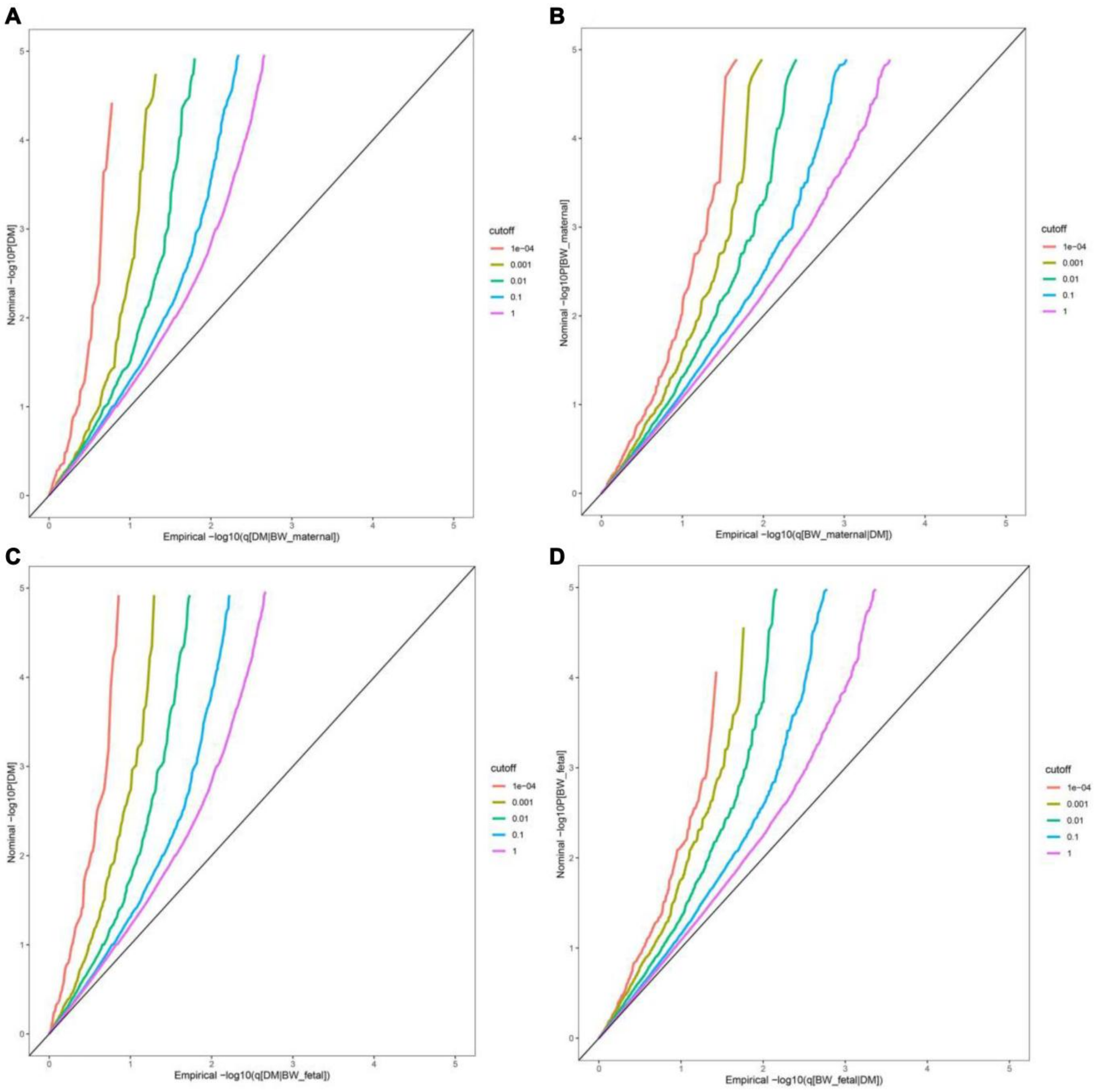

Supplementary Figure 2. Stratified Q-Q plots. Stratified Q-Q plots of nominal versus empirical -log10(*p*) values in T2D (**A**) as a function of the significance of the association with BW_maternal at the level of -log10(*p*) > 0, -log10(*p*) > 1, -log10(*p*) > 2, -log10(*p*) > 3 corresponding to $p \leq 1$, $p \leq 0.1$, $p \leq 0.01$, $p \leq 0.001$, and $p \leq 0.0001$, respectively. and (**B**) reversely BW_maternal as a function of the significance of the association with T2D. in (**C**) T2D as a function of significance of the association with BW_fetal; and in (**D**) BW_fetal as a function of significance with T2D.
