# Supplementary material for: Identification of novel functional CpG-SNPs associated with Type 2 diabetes and birth weight
Source: Aging (Albany NY). 2021 Apr 4;13(7):10619–58. doi: 10.18632/aging.202828 (PMC8064204; doi:10.18632/aging.202828)
Supplement: Supplementary Tables [file aging-13-202828-s003.pdf]

SUPPLEMENTARY TABLES

Supplementary Table 1. Conditional FDR value of 127 CpG-SNPs for T2DM given the BW (cFDR < 0.05)

Variant	chr	Pos	Alt	Gene	Annotation	Snp Type	Gene Type	eQTL/ meQTL/ metaQTL	*P*	cFDR
rs10449766	1	42070125	A/G	HNRNPFP1	28396 upstream	novel	novel		2.60E-03	4.99E-02
rs1415991	1	219625290	A/G	ZC3H11B	12145 upstream	novel	novel		8.60E-06	7.40E-03
rs340883	1	213972363	C/T	PROX1-AS1	non-coding intronic	confirmed	novel		3.80E-07	7.04E-05
rs7553890	1	213832562	T/C	PROX1-AS1	non-coding	novel	novel		2.20E-03	1.89E-02
rs1515114	2	226233671	A/G	AC062015.1	48.3kb upstream	novel	novel	eQTL	1.80E-04	8.17E-03
rs1522812	2	226132738	A/G	AC062015.1	47306 downstream	novel	novel	eQTL	9.30E-06	7.43E-04
rs2894593	2	226325601	T/C	AC062015.1	140230 upstream	novel	novel	eQTL	4.90E-04	9.16E-03
rs7605661	2	43397939	T/C	THADA	intronic	novel	confirmed	meQTL	1.30E-05	3.21E-04
rs12631028	3	12299907	C/T	PPARG	intronic	novel	confirmed	eQTL/meQTL	4.90E-05	4.81E-02
rs17013266	3	23449390	A/G	UBE2E2	intronic	novel	confirmed		1.90E-04	4.85E-02
rs17361324	3	123412407	C/T	ADCY5	intronic	novel	confirmed	meQTL	2.80E-09	8.40E-09
rs2290066	3	185728054	T/C	IGF2BP2, IGF2BP2-AS1	intronic, non-coding intronic	novel	confirmed	eQTL	4.90E-05	3.82E-02
rs4677887	3	123381376	T/G	ADCY5	intronic	confirmed	confirmed	eQTL	3.40E-06	6.80E-06
rs4677889	3	123424425	G/A	ADCY5	intronic	novel	confirmed		2.00E-03	2.53E-02
rs569255	3	125207090	G/A	SLC12A8	intronic	novel	novel	eQTL	1.70E-03	2.27E-02
rs6770420	3	170931960	G/A	KLF7P1	20890 downstream	novel	novel	eQTL/metaQTL	2.40E-05	1.75E-03
rs6794193	3	47073414	T/C	SETD2	intronic	novel	novel		6.60E-03	3.92E-02
rs6795735	3	64719689	C/T	ADAMTS9-AS2	non-coding intronic	confirmed	novel		2.10E-08	3.85E-05
rs720390	3	185830895	G/A	IGF2BP2	5839 upstream	confirmed	confirmed		1.40E-05	1.42E-02
rs9289218	3	123345984	C/T	ADCY5	intronic	novel	confirmed	eQTL	2.50E-03	1.60E-02
rs11097755	4	101788151	T/C	BANK1	intronic	novel	novel		1.60E-06	1.37E-03
rs1216373	4	128576009	C/T	AC078850.1	5478 upstream	novel	novel	metaQTL	6.60E-05	1.07E-02
rs3822109	4	75012761	C/T	PARM1, AC110760.1	coding nonsyn, non-coding intronic	novel	novel		3.20E-05	2.21E-02
rs6446490	4	6322920	G/A	PPP2R2C	3utr	confirmed	novel	eQTL/meQTL	1.70E-10	1.33E-06
rs7663887	4	17901297	C/A	LCORL	intronic	novel	novel	eQTL	8.80E-03	1.89E-02
rs10514870	5	59055501	A/G	PDE4D, AC092343.1	intronic, non-coding intronic	novel	novel		4.20E-03	2.18E-02
rs1650504	5	158602542	G/A	AC091939.1	13996 upstream	novel	novel		8.10E-05	9.88E-03
rs6867983	5	56558326	C/T	C5orf67	intronic	novel	confirmed		7.80E-06	9.07E-03
rs1012635	6	20675064	A/G	CDKAL1	intronic	confirmed	confirmed		4.40E-15	2.64E-14
rs12526403	6	41676676	C/T	TFEB	7302 downstream	novel	novel		1.90E-03	4.50E-02
rs1262557	6	126733443	C/T	RPS4XP9	49596 upstream	novel	novel		6.20E-05	8.80E-03
rs2206734	6	20694653	C/T	CDKAL1	intronic	novel	confirmed	meQTL	2.40E-27	9.60E-27
rs2307306	6	24781507	C/T	GMNN	coding nonsyn	novel	novel	eQTL	3.00E-05	1.82E-02
rs2745929	6	20754530	T/C	CDKAL1	intronic	novel	confirmed		4.50E-10	2.10E-09
rs4897378	6	130217352	C/T	SAMD3	5upstream, intronic	novel	novel	eQTL	4.80E-03	4.54E-02
rs6918311	6	136966564	A/G	RPL35AP3	7366 downstream	confirmed	novel	eQTL/meQTL	6.70E-07	1.16E-03
rs6941340	6	16037321	C/T	AL365265.1	4060 downstream	novel	novel		6.70E-05	4.75E-02
rs10244051	7	15024208	T/G	GTF3AP5	38.1kb upstream	novel	novel		2.40E-08	2.54E-05
rs17158736	7	111692378	G/C	DOCK4	33732 downstream	novel	novel		3.40E-05	3.89E-02
rs17689040	7	40880714	C/G	SUGCT	19951 upstream	novel	novel		2.70E-03	3.04E-02
rs6948511	7	27939096	T/C	JAZF1	intronic	novel	confirmed	metaQTL	5.70E-03	4.02E-02

rs7723	7	44578194	G/A	TMED4	3utr,3downstream	confirmed	novel	eQTL/metaQTL	2.50E-05	4.22E-04
rs7787720	7	13847029	C/T	AC005019.2	7326 downstream	confirmed	novel	eQTL/meQTL/ metaQTL	4.00E-06	3.66E-03
rs849135	7	28156794	G/A	JAZF1	intronic	confirmed	novel	eQTL/meQTL	6.60E-14	5.04E-10
rs1033129	8	138257741	T/C	FAM135B	intronic	novel	confirmed		4.70E-05	6.92E-03
rs2466311	8	117208857	G/C	SLC30A8	32143 upstream	novel	confirmed		1.20E-08	3.28E-05
rs7004862	8	94864735	T/G	INTS8	intronic	novel	confirmed	eQTL/metaQTL	5.50E-05	2.26E-03
rs7816345	8	36988591	C/T	AC090453.1	179 upstream	novel	novel	eQTL/metaQTL	2.30E-03	2.68E-02
rs10739970	9	94134010	A/G	PTPDC1	24154 upstream	novel	novel		9.90E-04	3.18E-02
rs10758593	9	4292083	G/A	GLIS3	intronic	confirmed	novel		2.90E-04	3.60E-02
rs10990568	9	95651855	A/G	AL354861.2	non-coding intronic	novel	novel		1.60E-03	2.46E-02
rs2383208	9	22132077	A/G	CDKN2B-AS1	10980 upstream	novel	confirmed	metaQTL	4.50E-13	2.77E-09
rs579459	9	133278724	T/C	ABO	3510 upstream	novel	confirmed	eQTL/meQTL/ metaQTL	8.50E-07	8.22E-06
rs7018475	9	22137686	T/G	CDKN2B-AS1	16589 upstream	novel	confirmed		7.20E-16	1.29E-11
rs10786044	10	92430823	A/G	MARK2P9	9948 upstream	novel	confirmed	eQTL/metaQTL	4.00E-05	1.36E-02
rs10885410	10	113064714	G/A	TCF7L2	intronic	novel	confirmed		1.60E-11	1.61E-07
rs11196229	10	113106413	G/A	TCF7L2	intronic	novel	confirmed		5.20E-09	2.70E-05
rs12245680	10	113060432	T/C	TCF7L2	intronic	confirmed	confirmed	meQTL	1.10E-10	7.90E-07
rs1225404	10	113154906	C/T	TCF7L2	intronic	novel	confirmed		1.80E-07	4.34E-04
rs1815314	10	79169036	G/A	ZMIZ1	intronic	novel	confirmed		2.90E-05	4.02E-02
rs1867567	10	68223370	G/A	ATOH7	7254 downstream	novel	novel		2.90E-04	4.87E-02
rs190925	10	69561187	G/A	NEUROG3	10511 downstream	novel	novel		4.20E-05	9.12E-03
rs2421019	10	122391070	C/T	PLEKHA1	5upstream	novel	confirmed	eQTL	3.50E-08	1.17E-06
rs2488071	10	92739820	A/G	Y_RNA	29212 upstream	confirmed	novel	eQTL/metaQTL	3.10E-11	1.96E-10
rs7070786	10	92363930	C/T	MARCH5	9966 upstream	novel	novel	eQTL	1.80E-06	9.54E-05
rs7899603	10	92465260	G/C	IDE	intronic	confirmed	confirmed	eQTL	3.80E-06	8.59E-04
rs7904519	10	113014168	A/G	TCF7L2	intronic	novel	confirmed	eQTL/meQTL	3.20E-38	2.00E-33
rs1002226	11	17384070	C/T	AC124798.1	0.5kb upstream	novel	novel	eQTL/meQTL	2.40E-06	2.28E-03
rs11037685	11	43857990	A/G	AC087521.2	non-coding intronic	novel	novel	eQTL	1.80E-06	3.08E-03
rs11819995	11	128519496	C/T	ETS1	intronic	novel	novel	meQTL	5.00E-05	2.86E-02
rs12786533	11	2875083	G/A	KCNQ1DN	2978 upstream	novel	novel	eQTL/meQTL	5.20E-05	4.34E-02
rs1447351	11	92984997	A/G	MTNR1B	3utr	novel	confirmed	metaQTL	8.20E-05	1.47E-03
rs151216	11	2659585	C/T	KCNQ1, KCNQ1OT1	intronic, non-coding	novel	comfirmed, novel		8.70E-07	1.33E-05
rs163171	11	2799835	T/C	KCNQ1	intronic	novel	confirmed	meQTL	8.90E-07	1.95E-03
rs163177	11	2817183	T/C	KCNQ1	intronic	confirmed	confirmed	eQTL	2.00E-12	2.07E-10
rs2237892	11	2818521	C/T	KCNQ1	intronic	novel	confirmed	meQTL/metaQ TL	1.10E-07	4.91E-05
rs231354	11	2685121	T/C	KCNQ1, KCNQ1OT1	intronic, non-coding	confirmed	comfirmed, novel	eQTL/meQTL	3.70E-06	2.40E-04
rs2334499	11	1675619	C/T	FAM99B	7650 downstream	confirmed	novel	eQTL/meQTL	7.30E-05	1.87E-02
rs234857	11	2831299	T/C	KCNQ1	intronic	confirmed	confirmed		2.00E-09	1.09E-06
rs3213225	11	2135306	G/A	IGF2,INS-IGF2	intronic,intronic	novel	confirmed	eQTL/meQTL	8.10E-04	4.15E-03
rs3852527	11	2805373	A/G	KCNQ1	intronic	confirmed	confirmed	eQTL	6.20E-06	1.02E-02
rs4937729	11	132833690	A/C	OPCML	intronic	novel	novel		1.30E-04	2.80E-02
rs1042725	12	65964567	C/T	HMGA2	3utr,3downstream	confirmed	confirmed	metaQTL	7.70E-04	1.54E-03
rs10774202	12	4168281	A/G	AC007207.1	50149 upstream	novel	novel		9.50E-04	2.42E-02
rs10774563	12	120622977	G/A	AC125616.1	6634 downstream	novel	novel	eQTL/metaQTL	3.30E-06	4.76E-03
rs10862960	12	77030355	C/T	E2F7	intronic	novel	novel	eQTL	1.70E-03	2.14E-02

rs10878353	12	65988752	T/C	HMGA2	2245 upstream	novel	confirmed		2.20E-03	5.34E-03
rs1169302	12	120994499	T/G	HNF1A	intronic	novel	novel	eQTL	2.60E-05	3.93E-02
rs12422899	12	133115743	T/C	ZNF891, AC026786.2	3utr, non-coding intronic	novel	novel, novel	eQTL/meQTL	4.90E-05	3.57E-02
rs192210	12	21615041	G/A	GYS2	10194 upstream	novel	novel	eQTL	1.10E-04	4.89E-02
rs3962536	12	22861176	A/G	AC084816.1	non-coding	novel	novel		1.50E-04	4.09E-02
rs4930718	12	123428886	A/G	RILPL2	intronic	novel	novel	eQTL	8.90E-04	6.05E-03
rs12865243	13	40104683	G/A	LINC00598	non-coding intronic	novel	novel		5.80E-03	2.44E-02
rs17202418	13	36163442	T/C	SOHLH2	4766 downstream	novel	novel	metaQTL	3.50E-05	1.31E-02
rs2066612	13	50171259	G/A	DLEU1	non-coding intronic	novel	novel	eQTL/metaQTL	2.70E-05	2.89E-02
rs4885692	13	80066087	C/T	AL158064.2	non-coding intronic	novel	novel		2.30E-05	1.90E-02
rs842379	13	46686202	C/T	LRCH1	intronic	novel	novel		1.60E-05	1.67E-02
rs9532498	13	40104306	G/C	LINC00598	non-coding intronic	novel	novel		2.80E-03	2.05E-02
rs10146260	14	76010803	G/T	IFT43	intronic	novel	novel		3.50E-05	1.57E-02
rs1285850	14	91419362	A/G	AL133153.2	non-coding intronic	confirmed	novel	eQTL	5.80E-05	4.61E-02
rs12894779	14	32835814	G/A	AKAP6	3utr	novel	confirmed		2.70E-04	2.05E-02
rs17117189	14	82713706	G/A	LINC02301	6881 upstream	novel	novel		1.20E-04	4.37E-02
rs11073964	15	91000531	C/T	VPS33B	coding nonsyn	novel	novel	eQTL/meQTL	2.10E-06	3.25E-03
rs1436955	15	62112183	C/T	NPM1P47	28878 upstream	novel	novel	eQTL	2.60E-05	2.87E-02
rs2682920	15	77577575	C/T	AC046168.2	non-coding intronic	novel	novel	eQTL	2.20E-05	1.02E-02
rs7497492	15	23782133	A/G	LOC107984793	intronic	novel	novel		1.90E-04	4.18E-02
rs4625714	16	55607701	C/T	LPCAT2	21031 upstream	novel	novel		1.90E-03	2.64E-02
rs7198093	16	85193257	A/G	GSE1	intronic	novel	novel		3.80E-05	3.09E-02
rs9940128	16	53766842	G/A	FTO	intronic	confirmed	confirmed		2.60E-22	5.61E-18
rs11651755	17	37739849	C/T	HNF1B	intronic	confirmed	confirmed		3.40E-08	2.27E-05
rs1402657	17	9886035	C/T	GLP2R	intronic	novel	confirmed	eQTL	4.50E-05	2.78E-02
rs1531798	17	78826049	A/G	USP36	intronic	novel	novel	eQTL	9.90E-05	7.84E-03
rs198542	17	50567176	G/A	CACNA1G	intronic	novel	novel		5.20E-04	9.15E-03
rs2290770	17	75834935	G/A	UNC13D	coding syn	novel	novel		3.80E-06	4.72E-03
rs2291725	17	48961770	T/C	GIP	coding nonsyn	novel	confirmed	eQTL/meQTL	7.10E-06	4.30E-03
rs390200	17	7206676	A/G	DLG4	intronic	novel	novel	eQTL	1.50E-02	1.88E-02
rs6565531	17	81049580	G/A	BAIAP2	intronic	novel	novel	eQTL/meQTL	3.90E-04	1.88E-02
rs878619	17	50555910	A/G	SPATA20	58 upstream	novel	novel	eQTL/meQTL	2.00E-04	2.23E-03
rs9303407	17	59299448	A/G	SNRPGP17	18105 upstream	novel	novel	eQTL	2.90E-04	3.20E-02
rs11659412	18	7063533	G/A	LAMA1	intronicc	novel	confirmed	metaQTL	1.70E-06	5.46E-03
rs7238644	18	29436831	C/T	AC074237.1	74868 upstream	novel	novel		4.30E-05	3.57E-02
rs1205438	20	38247165	C/T	KIAA1755	intronic	novel	novel	eQTL	1.10E-04	2.19E-02
rs2426778	20	58718421	G/A	NPEPL1	non-coding	novel	novel	eQTL	7.70E-03	4.76E-02
rs926345	20	41143307	T/C	PLCG1	intronic	novel	novel	eQTL/meQTL	5.70E-03	3.11E-02
rs11908784	21	19628605	A/G	AP000403.1	7060 upstream	novel	novel		5.80E-05	2.92E-02
rs137848	22	50001867	T/C	IL17REL	intronic	novel	novel	eQTL/meQTL	3.00E-06	3.55E-04
rs6006393	22	30194037	T/C	AC002378.1	non-coding intronic	confirmed	novel		2.00E-06	2.14E-04

Supplementary Table 2. meQTL associated effects of novel CpG-SNPs

CpG-SNP	Traits	Chr	Allele	Gene	Pos	meQTL Type	*P* value
rs7605661	Pleiotropic	2	T/C	THADA	43397939	cis	3.23E-29
rs17361324	Pleiotropic	3	C/T	ADCY5	123412407	cis	1.77E-38
rs6794193(LD to rs295442)	Pleiotropic	3	T/C	SETD2	47073414	cis	1.06E-33
rs6794193(LD to rs295458)	Pleiotropic	3	T/C	SETD2	47073414	cis	6.79E-21
rs6794193(LD to rs4078466)	Pleiotropic	3	T/C	SETD2	47073414	cis	3.27E-310
rs6794193(LD to rs6785790)	Pleiotropic	3	T/C	SETD2	47073414	cis	3.27E-310
rs2206734	Pleiotropic	6	C/T	CDKAL1	20694653	trans	1.71E-07
rs579459	Pleiotropic	9	T/C	ABO	133278724	cis	1.45E-09
rs163177	Pleiotropic	11	T/C	KCNQ1	2799835	cis	1.71E-58
rs231354	Pleiotropic	11	T/C	KCNQ1, KCNQ1OT1	2685121	cis	1.43E-29
rs231354(LD to rs462402)	Pleiotropic	11	T/C	KCNQ1, KCNQ1OT1	2685121	cis	4.17E-46
rs3213225	Pleiotropic	11	G/A	IGF2, INS-IGF2	2135306	cis	4.24E-07
rs6565531	Pleiotropic	17	G/A	BAIAP2	81049580	cis	3.27E-310
rs878619(LD to rs989128)	Pleiotropic	17	A/G	SPATA20	50555910	cis	1.28E-43
rs926345(LD to rs4297946)	Pleiotropic	20	T/C	PLCG1	41143307	trans	1.61E-14
rs137848(LD to rs137864)	Pleiotropic	22	T/C	IL17REL	50001867	cis	5.70E-24
rs137848(LD to rs5771222)	Pleiotropic	22	T/C	IL17REL	50001867	cis	9.01E-147
rs12631028(LD to rs12493718)	DM	3	C/T	PPARG	12299907	cis	7.39E-07
rs6446490	DM	4	G/A	PPP2R2C	6322920	cis	2.81E-16
rs6918311(LD to rs947733)	DM	6	A/G	RPL35AP3	136966564	cis	7.58E-110
rs7787720(LD to rs17167582)	DM	7	C/T	AC005019.2	13847029	cis	2.04E-22
rs849135	DM	7	G/A	JAZF1	28156794	trans	1.22E-07
rs12245680	DM	10	T/C	TCF7L2	113060432	cis	8.59E-05
rs7904519(LD to rs7077247)	DM	10	A/G	TCF7L2	113014168	cis	9.91E-09
rs1002226(LD to rs2074314)	DM	11	C/T	AC124798.1	17384070	cis	5.01E-05
rs11819995	DM	11	C/T	ETS1	128519496	cis	5.19E-05
rs12786533(LD to rs16924912)	DM	11	G/A	KCNQ1DN	2875083	cis	1.48E-28
rs2237892(LD to rs2283228)	DM	11	C/T	KCNQ1	2818521	cis	1.45E-10
rs2334499	DM	11	C/T	FAM99B	1675619	cis	4.05E-09
rs12422899(LD to rs11147248)	DM	12	T/C	ZNF891, AC026786.2	133115743	cis	2.13E-12
rs11073964	DM	15	C/T	VPS33B	91000531	cis	5.73E-05
rs2291725(LD to rs3895874)	DM	17	T/C	GIP	48961770	cis	1.62E-06
rs10807805(LD to rs4719646)	BW	7	CA/C,CG	AMZ1	2714216	cis	6.51E-17
rs2886070	BW	1	G/A	ARHGEF2	156004180	cis	2.43E-11
rs6687139	BW	1	A/G	LINC01681	170204121	cis	1.90E-05
rs12623454(LD to rs1869026)	BW	2	G/C	AC073257.1	120568721	cis	5.30E-08
rs2952769(LD to rs2464975)	BW	2	T/C	METTL21A	207629538	cis	1.55E-154
rs4853831	BW	2	T/C	MYT1L, AC093390.2	1809892	cis	1.60E-77
rs6918981	BW	6	G/A	AL354740.1	34270737	trans	2.57E-07
rs7766106(LD to rs9491706)	BW	6	C/T	RSPO3	127133993	cis	1.41E-44
rs2191883(LD to rs4720169)	BW	7	T/C	TBX20	35233679	cis	5.16E-06
rs855715	BW	10	G/T	ADRB1	114063765	cis	2.48E-06
rs4980661(LD to rs12365305)	BW	11	G/A	AP000439.2	69491811	cis	1.02E-04
rs12306172(LD to rs35786993)	BW	12	G/A	SMUG1, SMUG1-AS1	54145221	cis	7.72E-05
rs2293429(LD to rs2272300)	BW	12	A/C	CSAD	53180119	cis	7.73E-36

rs3184504	BW	12	T/C	SH2B3	111446804	cis	6.30E-07
rs3184504(LD to rs10774625)	BW	12	T/C	SH2B3	111446804	trans	4.10E-16
rs3184504(LD to rs653178)	BW	12	T/C	SH2B3	111446804	trans	9.62E-17
rs8039305	BW	15	T/C	FURIN	90879313	cis	3.63E-29
rs11079803	BW	17	G/A	PNPO	47942535	cis	5.25E-09
rs3760318	BW	17	G/A	ADAP2	30920697	cis	3.46E-05
rs4647887	BW	17	A/G	SNHG16	76562724	cis	6.23E-20
rs12455403	BW	18	T/C	EPB41L3	5620115	cis	2.22E-07
rs2261988	BW	19	G/T	UHRF1	4910877	cis	1.83E-05
rs2261988(LD to rs2602710)	BW	19	G/T	UHRF1	4910877	cis	1.85E-17
rs2261988(LD to rs3786942)	BW	19	G/T	UHRF1	4910877	cis	6.37E-231
rs2261988(LD to rs4544355)	BW	19	G/T	UHRF1	4910877	cis	1.55E-154
rs492602	BW	19	A/G	FUT2	48703160	trans	2.50E-07
rs492602(LD to rs281379)	BW	19	A/G	FUT2	48703160	trans	8.68E-08
rs492602(LD to rs503279)	BW	19	A/G	FUT2	48703160	trans	6.56E-08
rs492602(LD to rs504963)	BW	19	A/G	FUT2	48703160	cis	7.75E-10
rs492602(LD to rs601338)	BW	19	A/G	FUT2	48703160	cis	1.61E-222
rs492602(LD to rs602662)	BW	19	A/G	FUT2	48703160	trans	2.42E-08
rs492602(LD to rs681343)	BW	19	A/G	FUT2	48703160	cis	2.64E-257
rs5765275(LD to rs2272804)	BW	22	A/G	SMC1B	45352459	cis	3.27E-310
rs5765275(LD to rs5765335)	BW	22	A/G	SMC1B	45352459	cis	3.27E-310

Supplementary Table 3. metabolic QTL (mQTL) Effect of Novel CpG-SNPs

rsID	Traits	Metabolics	related disease or biofunctions (PMID)	Sample Type	*P*	Source	PMID
rs677042	Pleiotropic	ursodeoxycholate	Colorectal cancer (23940645)	serum	4.71E-05	SI data (Shin et al.)	24816252
rs6948511	Pleiotropic	X-11795		serum	3.30E-06	SI data (Shin et al.)	24816252
rs7723	Pleiotropic	1-oleoylglycerophosphocholine	T2D (2528830)	serum	2.12E-05	SI data (Shin et al.)	24816252
rs1042725	Pleiotropic	serine	Pancreatic cancer (20300169), Obesity (24740590)	serum	7.98E-07	SI data (Shin et al.)	24816252
rs1447351	Pleiotropic	X-13619		serum	4.98E-05	SI data (Shin et al.)	24816252
rs2488071	Pleiotropic	21-hydroxypregnenolone disulfate		serum	7.04E-07	SI data (Long et al.)	28263315
		leucine	Pancreatic cancer (20300169), Colorectal cancer (27275383)	serum	8.11E-05	SI data (Shin et al.)	24816252
rs579459	Pleiotropic	glycylglycine	Colorectal cancer (27275383), Alzheimer's disease (28951883)	serum	8.38E-10	SI data (Long et al.)	28263315
		phenylalanylserine	Colorectal cancer (27275383)	serum	2.78E-06	SI data (Long et al.)	28263315
		leucylalanine	Colorectal cancer (27275383), Pancreatic cancer (20300169), Uremia (22626821)	serum	8.62E-11	SI data (Long et al.)	28263315
		X-17178		serum	1.57E-09	SI data (Long et al.)	28263315
		alpha-glutamylglycine		serum	5.11E-09	SI data (Long et al.)	28263315
		X-14086		serum	5.15E-05	SI data (Shin et al.)	24816252
		ADpSGEGDFXAEGGGVR*	T2D (30372032)	serum	2.09E-19	SI data (Shin et al.)	24816252
		phenylalanylserine	Colorectal cancer (27275383)	serum	3.53E-07	SI data (Shin et al.)	24816252
		leucylalanine	Colorectal cancer (27275383), Pancreatic cancer (20300169), Uremia (22626821)	serum	1.26E-08	SI data (Shin et al.)	24816252
		alpha-glutamyltyrosine	Colorectal cancer (27275383)	serum	2.64E-05	SI data (Shin et al.)	24816252
		aspartylphenylalanine	Colorectal cancer (27275383)	serum	5.21E-07	SI data (Shin et al.)	24816252
		O-sulfo-L-tyrosine	CKD (26449609)	serum	3.03E-09	SI data (Shin et al.)	24816252
rs7004862	Pleiotropic	SM C24:0		serum	5.19E-05	SI data (Draisma et al.)	26068415
rs7816345	Pleiotropic	PC aa C34:4	Obesity (26910390)	serum	7.47E-05	SI data (Draisma et al.)	26068415
		hyodeoxycholate	Primary biliary cirrhosis (2621422)	serum	2.87E-05	SI data (Suhre et al.)	21886157
rs7787720	T2D	salicyluric glucuronide*		serum	9.33E-05	SI data (Shin et al.)	24816252
rs2383208	T2D	lysine	Pancreatic cancer (20300169), Colorectal cancer (20156336)	serum	8.79E-06	SI data (Shin et al.)	24816252
rs10786044	T2D	2-hydroxypalmitate	CKD (5672969)	serum	8.89E-05	SI data (Shin et al.)	24816252
rs2237892	T2D	gamma-glutamylvaline	Inflammatory Response (28691814)	serum	6.02E-05	SI data (Suhre et al.)	21886157
		N2,N2-dimethylguanosine	Kidney disease (9607216)	serum	9.42E-05	SI data (Shin et al.)	24816252

rs1216373	T2D	ergothioneine		serum	8.69E-05	SI data (Shin et al.)	24816252
rs10774563	T2D	butyrylcarnitine	Obesity (26910390)	serum	5.32E-121	SI data (Shin et al.)	24816252
		ethylmalonate	Anorexia nervosa (10197568), Malonyl-Coa decarboxylase deficiency (9177981)	serum	1.92E-62	SI data (Long et al.)	28263315
		butyrylcarnitine	Obesity (26910390)	serum	7.94E-50	SI data (Long et al.)	28263315
		methylsuccinate	Colorectal cancer (27275383)	serum	8.82E-19	SI data (Long et al.)	28263315
		butyrylcarnitine	Obesity (26910390)	serum	2.20E-16	SI data (Suhre et al.)	21886157
rs17202418	T2D	dihomo-linolenate (20:3n3 or n6)	oxidative stress (24760997)	serum	4.82E-05	SI data (Suhre et al.)	21886157
rs2066612	T2D	X-11847		serum	8.27E-05	SI data (Shin et al.)	24816252
rs11659412	T2D	X-23026		serum	9.85E-06	SI data (Long et al.)	28263315
		methylamine	Uremia (22626821), Crohn's disease (17269711)	urine	3.76E-05	SI data (Raffler et al.)	26352407
rs11125079	BW	HWESASXX*		serum	7.34E-05	SI data (Suhre et al.)	21886157
rs7701346	BW	X-11261		serum	4.81E-05	SI data (Shin et al.)	24816252
rs863818	BW	1-methylxanthine	Colorectal cancer (27275383)，Asthma (15537072)	serum	2.95E-05	SI data (Suhre et al.)	21886157
rs3750640	BW	phenylalanylserine	Colorectal cancer (27275383)	serum	1.85E-05	SI data (Shin et al.)	24816252
rs9416062	BW	2-hydroxyisobutyrate	Colorectal cancer (27275383)	urine	5.38E-05	SI data (Raffler et al.)	26352407
rs11051137	BW	X-24309		serum	7.93E-06	SI data (Long et al.)	28263315
rs3184504	BW	hypoxanthine	Colorectal cancer (27275383), Pancreatic cancer (20300169), Uremia (22626821)	serum	8.94E-07	SI data (Long et al.)	28263315
		C-glycosyltryptophan*		serum	1.66E-05	SI data (Shin et al.)	24816252
		gamma-glutamylleucine	Colorectal cancer (27275383)	serum	9.21E-05	SI data (Shin et al.)	24816252
		kynurenine	Crohn's disease (27609529), Ulcerative colitis (27609529)	serum	6.05E-18	SI data (Shin et al.)	24816252
		erythronate*	Colorectal cancer (27275383)	serum	8.62E-06	SI data (Shin et al.)	24816252
		laurate (12:0)		serum	8.19E-05	SI data (Suhre et al.)	21886157
rs7296248	BW	glycodeoxycholate	Colorectal cancer (27275383)	serum	7.18E-05	SI data (Shin et al.)	24816252
rs2586211	BW	allantoin	Crohn's disease (27609529), Colorectal cancer (25037050)	serum	5.80E-05	SI data (Shin et al.)	24816252
rs492602	BW	ADpSGEGDFXAEGGGVR*	T2D (30372032)	serum	2.62E-11	SI data (Shin et al.)	24816252
		X-08402		serum	2.03E-05	SI data (Shin et al.)	24816252
		citrulline	Crohn's disease (27609529), Gout (28270806)	serum	7.45E-05	SI data (Shin et al.)	24816252
rs533318		tiglyl carnitine		serum	4.81E-05	SI data (Suhre et al.)	21886157

Supplementary Table 4. metabolic pathway analysis ($P < 0.05$)

Traits	Pathway	Total	Hits	Impact	*P*-value	Details
T2D	Aminoacyl-tRNA biosynthesis	75	4	0.11268	9.15E-04	KEGG
	alpha-Linolenic acid metabolism	29	2	0.20335	1.34E-02	KEGG
	Methane metabolism	34	2	0.01778	1.81E-02	KEGG
	Glycerophospholipid metabolism	39	2	0.1037	2.35E-02	KEGG
BW	Aminoacyl-tRNA biosynthesis	75	3	0.05634	8.26E-03	KEGG
	Glycerophospholipid metabolism	39	2	0.1037	2.06E-02	KEGG

Supplementary Table 5. Conditional FDR value of 188 CpG-SNPs for BW given the T2DM (cFDR < 0.05)

Variant	Chr	Pos	Alt	Gene	Annotation	Snp Type	Gene Type	eQTL/meQTL/ metaQTL	*P*	cFDR
rs10449766	1	42070125	A/G	HNRNPFP1	28396 upstream	novel	novel		2.00E-03	3.73E-02
rs11264298	1	155036782	G/A	DCST1	intronic	novel	novel	eQTL	6.80E-05	3.07E-02
rs11537641	1	42930743	G/A	SLC2A1	coding syn	novel	novel		4.90E-05	4.43E-02
rs11589239	1	227849577	C/T	PRSS38	3107 upstream	novel	novel	eQTL	5.80E-04	3.77E-02
rs1415181	1	214857808	T/C	GAPDHP24	12926 downstream	novel	novel		3.70E-06	5.20E-03
rs2744718	1	22197234	T/C	WNT4	53265 upstream	novel	novel	eQTL	3.50E-06	4.00E-03
rs2886070	1	156004180	G/A	ARHGEF2	intronic	novel	novel	eQTL/meQTL	3.50E-08	3.39E-05
rs340883	1	213972363	C/T	PROX1-AS1	non-coding intronic	novel	novel		7.10E-03	2.13E-02
rs6687139	1	170204121	A/G	LINC01681	non-coding intronic	novel	novel	eQTL/meQTL	3.80E-05	3.54E-02
rs7527321	1	232632149	T/C	RNU6-1211P	68055 downstream	novel	novel		1.30E-05	1.64E-02
rs7542242	1	22151000	C/T	WNT4	7031 upstream	novel	novel	eQTL	3.80E-05	3.62E-02
rs7553890	1	213832562	T/C	PROX1-AS1	non-coding	novel	novel		6.70E-05	2.53E-03
rs10165908	2	157459117	T/C	CYTIP	intronic	novel	novel		5.40E-05	3.17E-02
rs11125079	2	46505076	C/T	ATP6V1E2	non-coding intronic	novel	novel	eQTL/metaQTL	9.50E-06	1.04E-02
rs12476224	2	56154789	A/G	AC011306.1, AC007744.1	non-coding intronic, non-coding intronic	novel	novel		2.40E-04	3.82E-02
rs12623454	2	120568721	G/C	AC073257.1	10612 upstream	novel	novel	meQTL	2.40E-05	1.66E-02
rs1515114	2	226233671	A/G	AC062015.1	48300 upstream	novel	novel	eQTL	2.60E-03	1.82E-02
rs1522812	2	226132738	A/G	AC062015.1	47306 downstream	novel	novel	eQTL	4.30E-03	1.55E-02
rs17745230	2	46263191	C/T	Metazoa_SRP	3499 downstream	novel	novel	eQTL	9.10E-07	1.01E-03
rs1901263	2	46591447	C/T	PIGF	intronic	novel	novel	eQTL	3.50E-05	1.21E-02
rs2894593	2	226325601	T/C	AC062015.1	140230 upstream	novel	novel	eQTL	3.80E-04	6.05E-03
rs2952769	2	207629538	T/C	METTL21A	3610 upstream	novel	novel	eQTL/meQTL	8.50E-06	8.79E-03
rs34367192	2	9502349	C/T	ADAM17	intronic	novel	novel	eQTL	2.50E-04	1.29E-02
rs4669521	2	10051809	G/A	KLF11	intronic	novel	novel	eQTL	6.20E-05	4.91E-02
rs4853831	2	1809892	T/C	MYT1L, AC093390.2	intronic, non-coding	novel	novel	eQTL/meQTL	5.10E-05	2.71E-02
rs6735418	2	43002048	A/G	AC016735.1	non-coding intronic	novel	novel	eQTL	1.00E-05	1.34E-02
rs7561273	2	24024644	A/G	MFSD2B	intronic	novel	novel	eQTL	2.10E-07	2.56E-04
rs7605661	2	43397939	T/C	THADA	intronic	novel	novel	meQTL	3.90E-04	1.87E-03
rs935172	2	26581379	T/C	CIB4	coding nonsyn	novel	novel		9.30E-05	3.71E-02
rs17290714	3	47854215	C/T	MAP4	intronic	novel	novel	eQTL	1.80E-05	1.87E-02
rs17361324	3	123412407	C/T	ADCY5	intronic	novel	novel	meQTL	8.00E-20	1.20E-18
rs2306531	3	157099873	C/T	LINC00880, LINC00881	non-coding intronic, 3downstream	novel	novel	eQTL	8.60E-06	1.34E-02
rs4677887	3	123381376	T/G	ADCY5	intronic	confirmed	confirmed	eQTL	2.30E-12	2.91E-11

rs4677889	3	123424425	G/A	ADCY5	intronic	novel	novel		4.50E-04	1.02E-02
rs4681161	3	148891258	T/C	AC092979.1, CPA3	non-coding intronic, intronic	novel	novel	eQTL	4.40E-09	3.45E-06
rs569255	3	125207090	G/A	SLC12A8	intronic	novel	novel	eQTL	4.50E-04	1.01E-02
rs6770420	3	170931960	G/A	KLF7P1	20890 downstream	novel	novel	eQTL/metaQTL	4.60E-03	1.63E-02
rs6794193	3	47073414	T/C	SETD2	intronic	novel	novel	eQTL/meQTL	4.20E-05	3.02E-03
rs870429	3	123159172	A/G	PDIA5	intronic	novel	novel	eQTL	1.20E-04	2.42E-02
rs900399	3	157080943	A/G	LINC00880	724 downstream	novel	novel	eQTL	2.90E-41	4.05E-37
rs9289218	3	123345984	C/T	ADCY5	intronic	novel	novel	eQTL	1.80E-05	8.91E-04
rs10020719	4	129331259	A/G	AC082650.1	26237 upstream	novel	novel		5.00E-05	2.21E-02
rs1129998	4	150798111	A/G	LRBA	coding syn	novel	novel		1.00E-05	9.19E-03
rs12502033	4	144701499	T/C	HHIP, GYPA	intron, intron	novel	novel		1.10E-07	1.39E-04
rs17447835	4	42592928	A/G	ATP8A1	intronic	novel	novel	eQTL	8.00E-05	4.72E-02
rs2301718	4	105088606	G/A	AC096577.1	non-coding intronic	novel	novel		6.60E-05	4.59E-02
rs7663887	4	17901297	C/A	LCORL	intronic	novel	novel	eQTL	2.70E-09	5.90E-07
rs7682893	4	130355629	G/A	LINC02479	20600 downstream	novel	novel		2.60E-05	2.68E-02
rs10514870	5	59055501	A/G	PDE4D, AC092343.1	intron, non-coding intronic	novel	novel		5.00E-06	4.17E-04
rs110873	5	57632548	A/G	AC008780.2	15117 upstream	novel	novel	eQTL	3.60E-05	3.52E-02
rs12653511	5	77014955	A/G	AGGF1	14296 downstream	novel	novel		3.00E-05	2.94E-02
rs13153101	5	68289314	A/C	PIK3R1	intronic	novel	novel		5.00E-06	7.86E-03
rs1705392	5	67204440	C/G	CD180	7641 upstream	novel	novel		6.90E-04	3.55E-02
rs4130707	5	85071655	G/T	AC114928.1	40687 downstream	novel	novel		9.50E-05	4.75E-02
rs4699908	5	57618434	G/A	AC008780.2	1003 upstream	novel	novel		4.50E-06	3.46E-03
rs6556350	5	158452481	C/T	AC091979.1	non-coding	novel	novel		2.30E-06	2.55E-03
rs7701346	5	134492665	A/G	AC005355.1	146 upstream	novel	novel	eQTL/metaQTL	4.20E-04	2.43E-02
rs7717779	5	8429660	C/T	LINC02226, C091965.1	non-coding intronic, non-coding intronic	novel	novel		3.40E-05	4.17E-03
rs845734	5	109687709	C/A	AC012603.1	93 downstream	novel	novel	eQTL	2.00E-05	1.36E-02
rs863818	5	68258195	A/G	PIK3R1	intronic	novel	novel	metaQTL	1.10E-05	1.22E-02
rs10080410	6	132289467	G/A	MOXD1	6588 downstream	novel	novel		8.60E-04	2.81E-02
rs1012635	6	20675064	A/G	CDKAL1	intronic	confirmed	confirmed		4.00E-10	1.00E-09
rs1040525	6	142382532	C/T	ADGRG6	intronic	novel	novel	eQTL	6.10E-05	4.91E-02
rs10946101	6	165751179	C/G	PDE10A	non-coding intronic	novel	novel		7.20E-04	2.46E-02
rs10947463	6	33879300	G/A	LINC01016	non-coding intronic	novel	novel	eQTL	2.20E-05	2.04E-02
rs10947659	6	37141909	C/A	PIM1	28294 downstream	novel	novel		1.80E-05	2.05E-02
rs12526403	6	41676676	C/T	TFEB	7302 downstream	novel	novel		2.10E-03	3.83E-02
rs1361024	6	151749793	G/A	ESR1	intronic	novel	novel		8.00E-06	1.25E-02
rs1415701	6	130024690	G/A	L3MBTL3	intronic	confirmed	novel	eQTL	4.00E-11	3.30E-07
rs1547669	6	33807864	A/G	MLN	3853 upstream	novel	novel	eQTL	1.00E-05	5.28E-03
rs2206734	6	20694653	C/T	CDKAL1	intronic	novel	novel	meQTL	2.40E-17	4.80E-17
rs2273669	6	108963986	A/G	ARMC2	intronic	novel	novel	eQTL	7.60E-06	1.07E-02
rs2745929	6	20754530	T/C	CDKAL1	intronic	novel	novel		1.10E-09	4.77E-09
rs2982570	6	151692613	C/T	ESR1	intronic	novel	novel		4.30E-07	3.38E-04
rs4897378	6	130217352	C/T	SAMD3	5upstream	novel	novel	eQTL	2.80E-04	1.20E-02
rs6918981	6	34270737	G/A	AL354740.1	non-coding intronic	novel	novel	eQTL/meQTL	1.10E-05	5.44E-03
rs6933511	6	130135793	A/C	L3MBTL3, KLF7P1	intronic, non-coding intronic	novel	novel	eQTL	2.60E-06	1.71E-03
rs7766106	6	127133993	C/T	RSPO3	intronic	novel	novel	eQTL/meQTL	2.60E-05	1.48E-02
rs9385532	6	130050082	T/C	L3MBTL3	intronic	novel	novel	eQTL	2.00E-04	4.18E-02

rs9492469	6	130169033	G/A	SAMD3	intronic	novel	novel	eQTL	1.80E-05	2.01E-02
rs10807805	7	2714216	CA/C,CG	AMZ1	3utr	novel	novel	eQTL/meQTL	1.40E-07	4.61E-04
rs12704091	7	149267936	A/G	ZNF783	intronic	novel	novel	eQTL	9.70E-07	1.10E-03
rs17401675	7	73643220	A/G	MLXIPL	18677 upstream	novel	confirmed	eQTL	1.50E-11	2.12E-08
rs17689040	7	40880714	C/G	SUGCT	19951 upstream	novel	novel		6.20E-04	1.31E-02
rs2191883	7	35233679	T/C	TBX20	intronic	novel	novel	meQTL	4.20E-06	7.90E-03
rs2389995	7	18933395	A/G	HDAC9	intronic	novel	novel		6.30E-05	3.34E-02
rs4143341	7	159262263	A/G	PIP5K1P2	28886 upstream	novel	novel	eQTL	3.20E-06	6.93E-03
rs6947302	7	149172403	T/C	ZNF398	intronic	novel	novel		5.40E-04	4.31E-02
rs6948511	7	27939096	T/C	JAZF1	intronic	novel	novel	metaQTL	9.50E-05	5.80E-03
rs7723	7	44578194	G/A	TMED4	3utr	novel	novel	eQTL/metaQTL	1.40E-04	8.40E-04
rs12677785	8	141243157	A/G	SLC45A4	intronic	novel	novel	eQTL	2.70E-05	1.20E-02
rs17217757	8	105601093	G/C	ZFPM2	intronic	novel	novel		5.90E-05	3.12E-02
rs6989280	8	125496504	G/A	AC091114.1	non-coding intronic	confirmed	novel		5.00E-08	1.10E-04
rs7004862	8	94864735	T/G	INTS8	intronic	novel	novel	eQTL/metaQTL	1.70E-03	9.35E-03
rs7816345	8	36988591	C/T	AC090453.1	179 upstream	novel	novel	eQTL/metaQTL	4.20E-04	1.05E-02
rs10739970	9	94134010	A/G	PTPDC1	24154 upstream	novel	novel		3.00E-03	3.78E-02
rs10816736	9	108835654	C/T	AL359692.1	679 downstream	novel	novel	eQTL	1.40E-04	3.67E-02
rs10990568	9	95651855	A/G	AL354861.2	non-coding intronic	novel	novel		5.30E-04	1.19E-02
rs2000244	9	123211138	A/G	STRBP	intronic	novel	novel	eQTL	1.80E-10	2.33E-07
rs2236407	9	95475514	A/G	PTCH1	intronic	novel	novel	eQTL	1.70E-08	2.48E-05
rs473902	9	95493953	T/G	PTCH1	intronic	novel	novel		6.70E-07	1.49E-03
rs534643	9	111276424	C/T	AL162414.1	non-coding intronic	novel	novel		1.20E-04	4.35E-02
rs579459	9	133278724	T/C	ABO	3510 upstream	novel	novel	eQTL/meQTL/ metaQTL	8.30E-06	3.60E-05
rs9721852	9	89748077	T/C	UNQ6494	28318 upstream	novel	novel		1.10E-05	1.39E-02
rs11250238	10	1043676	T/C	IDI2-AS1/IDI1	intron,intron	novel	novel	eQTL	4.00E-06	1.80E-03
rs1638410	10	116766015	T/C	HSPA12A	intronic	novel	novel		5.70E-05	3.56E-02
rs17566087	10	69219200	A/G	AL596223.1	non-coding intronic	novel	novel	eQTL	2.30E-05	2.50E-02
rs2421019	10	122391070	C/T	PLEKHA1	5upstream,intronic	novel	novel	eQTL	2.70E-04	9.45E-04
rs2488071	10	92739820	A/G	Y_RNA	29212 upstream	novel	novel	eQTL/metaQTL	2.70E-08	9.00E-08
rs3750640	10	5654519	G/A	ASB13	intronic	novel	novel	eQTL/metaQTL	3.00E-04	3.08E-02
rs7070786	10	92363930	C/T	5-Mar	9966 upstream	novel	novel	eQTL	1.50E-03	4.80E-03
rs7088711	10	102799509	G/A	WBP1L	intronic	novel	novel	eQTL	4.50E-05	5.71E-03
rs855715	10	114063765	G/T	ADRB1	16857 upstream	novel	novel	meQTL	1.50E-07	1.65E-04
rs9416062	10	75490730	A/G	LRMDA	intronic	novel	novel	eQTL/metaQTL	3.40E-04	3.70E-02
rs10840346	11	10041452	G/A	SBF2	intronic	novel	novel	eQTL	7.00E-07	7.60E-04
rs1447351	11	92984997	A/G	MTNR1B	3utr	novel	novel	metaQTL	2.30E-04	2.05E-03
rs151216	11	2659585	C/T	KCNQ1, KCNQ1OT1	intronic, non-coding	novel	novel		5.10E-05	1.97E-04
rs163177	11	2817183	T/C	KCNQ1	intronic	novel	novel	eQTL/meQTL	5.70E-04	1.52E-03
rs1944055	11	58575156	A/G	LPXN	intronic	novel	novel		3.80E-04	3.97E-02
rs231354	11	2685121	T/C	KCNQ1, KCNQ1OT1	intron, non-coding	confirmed	confirmed	eQTL/meQTL	2.80E-03	9.10E-03
rs234857	11	2831299	T/C	KCNQ1	intronic	novel	novel		2.00E-02	4.67E-02
rs3213225	11	2135306	G/A	IGF2, INS-IGF2	intronic, intronic	novel	novel	eQTL/meQTL	2.70E-06	9.59E-05
rs4980661	11	69491811	G/A	AP000439.2	11871 upstream	novel	novel	meQTL	1.00E-04	1.98E-02
rs546240	11	30512075	C/T	MPPED2	intronic	novel	novel	eQTL	4.40E-05	3.71E-02
rs6590039	11	124071072	G/T	OR10D5P	15365 upstream	novel	novel		2.20E-05	2.40E-02

rs936370	11	81264293	C/T	MTND4LP18	287933 downstream	novel	novel		3.10E-04	4.26E-02
rs1042725	12	65964567	C/T	HMGA2	3utr	confirmed	confirmed	metaQTL	7.10E-32	1.90E-29
rs10774202	12	4168281	A/G	AC007207.1	50149 upstream	novel	novel		1.60E-03	2.32E-02
rs10862960	12	77030355	C/T	E2F7	intronic	novel	novel	eQTL	2.50E-04	7.30E-03
rs10878353	12	65988752	T/C	HMGA2	22457 upstream	novel	novel		1.40E-08	1.04E-06
rs10878359	12	66010844	T/C	MIR6074	12776 downstream	novel	novel		4.90E-10	1.36E-06
rs11051137	12	30884947	G/A	AC010198.2	22768 upstream	novel	novel	metaQTL	6.80E-05	4.50E-02
rs11067591	12	115429745	T/C	AC078880.2	65000 upstream	novel	novel	eQTL	5.80E-04	1.84E-02
rs12306172	12	54145221	G/A	SMUG1, SMUG1-AS1	intronic, non-coding intronic	novel	novel	eQTL/meQTL	3.80E-06	4.75E-03
rs12828089	12	46210774	C/A,T	SLC38A1	intronic	novel	novel	eQTL	3.70E-05	3.48E-02
rs1870566	12	65818237	T/C	RPSAP52	intronic	novel	novel		7.50E-06	4.29E-03
rs2293429	12	53180119	A/C	CSAD	intronic	novel	novel	eQTL/meQTL	5.00E-05	3.16E-02
rs3184504	12	111446804	T/C	SH2B3	coding nonsyn	novel	novel	eQTL/meQTL/meta QTL	3.70E-06	1.75E-03
rs4930718	12	123428886	A/G	RILPL2	intronic	novel	novel	eQTL	1.10E-05	3.28E-04
rs703545	12	102549222	A/G	AC010202.1	35567 upstream	novel	novel		8.70E-06	5.74E-03
rs7296248	12	102683420	C/T	LINC00485	125860 downstream	novel	novel	metaQTL	3.20E-04	4.49E-02
rs7961772	12	26798318	C/T	ITPR2	intronic	novel	confirmed	eQTL	3.80E-07	7.43E-04
rs7965495	12	66037910	G/A	RPL21P18	196 upstream	novel	novel		1.10E-04	2.97E-02
rs12865243	13	40104683	G/A	LINC00598	non-coding intronic	novel	novel		3.00E-06	3.32E-04
rs452674	13	40080917	T/C	LINC00598	non-coding	novel	novel		8.50E-05	1.70E-02
rs7331478	13	27919720	T/G	PDX1	300 downstream	novel	novel		6.50E-05	4.79E-02
rs9532498	13	40104306	G/C	LINC00598	non-coding intronic	novel	novel		5.20E-05	2.22E-03
rs4965425	15	98638434	C/T	AC118658.1	8517 downstream	novel	novel		6.40E-07	5.30E-04
rs8039305	15	90879313	T/C	FURIN	intronic	novel	novel	eQTL/meQTL	4.90E-08	4.55E-05
rs13331339	16	20179928	C/T	AC092132.2	21537 upstream	novel	novel		1.50E-04	3.79E-02
rs2397775	16	55718812	A/G	CES1P2	9303 downstream	novel	novel		2.40E-05	2.54E-02
rs2521477	16	20016691	T/C	GPR139	14794 downstream	novel	confirmed	eQTL	4.70E-07	1.33E-03
rs4625714	16	55607701	C/T	LPCAT2	21031 upstream	novel	novel		3.70E-04	1.05E-02
rs908382	16	27204059	A/C	KDM8	intronic	novel	novel		5.20E-05	2.91E-02
rs9938631	16	67397901	C/T	ZDHHC1	intronic	novel	novel	eQTL	2.50E-05	1.67E-02
rs11079803	17	47942535	G/A	PNPO	intronic	novel	novel	eQTL/meQTL	9.70E-06	5.09E-03
rs1215	17	7260031	A/G	AC003688.1, CLDN7	intronic,3utr	novel	novel	eQTL	1.20E-04	4.53E-02
rs12939237	17	49067202	G/A	IGF2BP1	11552 upstream	novel	novel	eQTL	1.80E-04	1.45E-02
rs1531798	17	78826049	A/G	USP36	intronic	novel	novel	eQTL	8.90E-03	3.72E-02
rs198542	17	50567176	G/A	CACNA1G	intronic	novel	novel		4.50E-04	6.42E-03
rs34870220	17	7181592	C/T	ASGR1	2028 upstream	novel	novel	eQTL	9.90E-07	7.32E-04
rs3760318	17	30920697	G/A	ADAP2	5upstream	novel	novel	eQTL/meQTL	7.90E-05	1.26E-02
rs390200	17	7206676	A/G	DLG4	intronic	novel	novel	eQTL	1.20E-12	6.89E-10
rs4647887	17	76562724	A/G	SNHG16	non-coding intronic	novel	novel	eQTL/meQTL	2.80E-04	4.93E-02
rs4793636	17	50062136	G/A	ITGA3	intronic	novel	novel	eQTL	4.10E-05	3.82E-02
rs6565531	17	81049580	G/A	BAIAP2	intronic	novel	novel	eQTL/meQTL	3.50E-03	3.61E-02
rs878619	17	50555910	A/G	SPATA20	58 upstream	novel	novel	eQTL/meQTL	2.80E-05	4.24E-04
rs12455403	18	5620115	T/C	EPB41L3	intronic	novel	novel	meQTL	3.80E-05	2.32E-02
rs2586211	18	11872826	G/A	GNAL	intronic	novel	novel	metaQTL	4.00E-05	1.38E-02
rs4798774	18	932568	G/A	LINC01904	4575 upstream	novel	novel		2.80E-04	4.94E-02
rs10113	19	46609391	T/C	CALM3, AC093503.2	utr-3, non-coding intronic	novel	novel		1.20E-03	3.79E-02

rs2261988	19	4910877	G/T	UHRF1	5utr	novel	novel	eQTL/meQTL	7.10E-06	3.39E-03
rs34033973	19	40437876	A/G	SERTAD3	2968 downstream	novel	novel	eQTL	5.90E-05	4.70E-02
rs492602	19	48703160	A/G	FUT2	coding syn	novel	novel	eQTL/meQTL/meta QTL	4.10E-07	5.10E-04
rs533318	19	40161784	T/C	MAP3K10	29960 downstream	novel	novel	eQTL/metaQTL	1.80E-04	4.94E-02
rs8182579	19	33418945	C/T	PEPD	intronic	novel	confirmed	eQTL	1.80E-05	1.12E-02
rs17536052	20	10664057	G/A	JAG1	intronic	novel	novel		7.80E-07	1.27E-03
rs1886843	20	58669145	A/G	STX16, STX16-NPEPL1	intronic, intronic	novel	novel	eQTL	2.00E-04	2.74E-02
rs2426778	20	58718421	G/A	NPEPL1	non-coding	novel	novel	eQTL	9.70E-05	6.10E-03
rs6016377	20	40544088	C/T	MAFB	141760 downstream	confirmed	confirmed		3.60E-10	2.24E-06
rs6057610	20	32653587	T/C	C20orf203	5upstream	novel	novel	eQTL	3.00E-10	1.99E-06
rs6075924	20	22531891	T/C	LINC00261	15780 downstream	novel	novel		4.80E-06	5.87E-03
rs6077888	20	10712341	C/T	AL050403.2	non-coding intronic	novel	novel		8.00E-08	6.47E-05
rs8125378	20	31852354	G/A	DUSP15	intronic	novel	novel	eQTL	4.10E-05	3.57E-02
rs926345	20	41143307	T/C	PLCG1	intronic	novel	novel	eQTL/meQTL	1.10E-05	9.32E-04
rs137848	22	50001867	T/C	IL17REL	intronic	novel	novel	eQTL/meQTL	7.50E-03	2.08E-02
rs5765275	22	45352459	A/G	SMC1B	coding syn	novel	novel	eQTL/meQTL	9.20E-06	1.16E-02
rs6006393	22	30194037	T/C	AC002378.1	non-coding intronic	novel	novel	eQTL	5.10E-03	1.53E-02

Supplementary Table 6. Significant results in LocusCompare analysis

GeneID	Gene Symbol	Chr	TSS	GWAS -log10(P)	eQTL -log10(P)	Traits
ENSG00000169047.5	IRS1	chr2	227596032	8.921	10.756	T2D
ENSG00000272622.1	RP11-395N3.2	chr2	227664861	8.921	8.641	T2D
ENSG00000153814.7	JAZF1	chr7	27870191	13.523	8.065	T2D
ENSG00000149084.7	HSD17B12	chr11	43577985	8.276	87.748	T2D
ENSG00000246250.2	RP11-613D13.5	chr11	43851258	8.276	11.800	T2D
ENSG00000107679.10	PLEKHA1	chr10	124134211	11.745	7.278	T2D
ENSG00000260196.1	RP1-239B22.5	chr11	17402195	7.367	10.564	T2D
ENSG00000188211.4	NCR3LG1	chr11	17373272	7.367	9.621	T2D
ENSG00000157895.7	C12orf43	chr12	121440315	7.444	26.424	T2D
ENSG00000153774.4	CFDP1	chr16	75327595	10.432	24.336	T2D
ENSG00000227117.2	CTA-85E5.10	chr22	30404730	8.409	15.734	T2D
ENSG00000163257.6	DCAF16	chr4	17802277	15.276	20.505	BW
ENSG00000254135.1	RP11-32D16.1	chr5	157912197	7.886	9.454	BW
ENSG00000146535.9	GNA12	chr7	2767745	7.886	8.225	BW
ENSG00000106635.3	BCL7B	chr7	72950685	10.237	12.136	BW
ENSG00000029534.15	ANK1	chr8	41510738	10.886	8.513	BW
ENSG00000226752.3	PSMD5-AS1	chr9	123587105	8.066	140.174	BW
ENSG00000213277.3	MARCKSL1P1	chr10	104935310	7.456	9.845	BW
ENSG00000107679.10	PLEKHA1	chr10	124134211	7.745	7.278	BW
ENSG00000182511.7	FES	chr15	91426924	7.770	10.397	BW
ENSG00000181885.14	CLDN7	chr17	7163259	15.041	16.767	BW
ENSG00000175826.7	CTDNEP1	chr17	7146909	15.041	9.632	BW
ENSG00000170291.10	ELP5	chr17	7155400	15.041	14.102	BW
ENSG00000264920.1	RP11-6N17.4	chr17	45968620	7.337	18.426	BW
ENSG00000175730.7	BAK1P1	chr20	31276722	11.114	13.738	BW
ENSG00000183762.8	KREMEN1	chr22	29469065	8.000	22.178	BW

Supplementary Table 7. Gene ontology (GO) terms enriched for SNP-annotated genes with FDR ≤ 0.05

Traits	Pathway ID	Pathway description	Gene count	FDR
T2DM	GO:0097110	scaffold protein binding	6	9.62E-04
	GO:0010817	regulation of hormone levels	13	3.04E-03
	GO:0050796	regulation of insulin secretion	8	4.19E-03
	GO:0032409	regulation of transporter activity	9	4.43E-03
	GO:0046883	regulation of hormone secretion	9	4.95E-03
	GO:0031016	pancreas development	6	5.32E-03
	GO:0009746	response to hexose	7	5.56E-03
	GO:0090276	regulation of peptide hormone secretion	8	5.67E-03
	GO:0009749	response to glucose	7	5.96E-03
	GO:0034284	response to monosaccharide	7	6.11E-03
	GO:2001257	regulation of cation channel activity	7	6.74E-03
	GO:0032412	regulation of ion transmembrane transporter activity	8	6.92E-03
	GO:0051049	regulation of transport	21	6.93E-03
	GO:1901700	response to oxygen-containing compound	19	7.33E-03
	GO:0022898	regulation of cation transmembrane transport	9	7.42E-03
	GO:0022898	regulation of transmembrane transporter activity	8	7.43E-03
	GO:0048878	chemical homeostasis	16	7.44E-03
	GO:0032879	regulation of localization	26	8.59E-03
	GO:0009743	response to carbohydrate	7	8.70E-03
	GO:0034762	regulation of transmembrane transport	11	8.87E-03
	GO:0044057	regulation of system process	11	9.81E-03
	GO:0051046	regulation of secretion	13	1.11E-02
	GO:0034765	regulation of ion transmembrane transport	10	1.12E-02
	GO:0031018	endocrine pancreas development	4	1.92E-02
	GO:1903530	regulation of secretion by cell	12	2.21E-02
	GO:0001890	placenta development	6	2.35E-02
	GO:0042592	homeostatic process	18	3.29E-02
	GO:0050708	regulation of protein secretion	9	4.67E-02
BW	GO:0005515	protein binding	109	7.86E-04
	GO:0097110	scaffold protein binding	7	1.18E-03
	GO:0003674	molecular_function	136	4.33E-03
	GO:0008134	transcription factor binding	17	4.92E-03
	GO:0005158	insulin receptor binding	4	1.96E-02
	GO:0009653	anatomical structure morphogenesis	33	2.56E-02
	GO:1901653	cellular response to peptide	11	2.57E-02
	GO:1901652	response to peptide	13	2.58E-02
	GO:0071417	cellular response to organonitrogen compound	14	2.58E-02
	GO:0033500	carbohydrate homeostasis	9	2.67E-02
	GO:0045893	positive regulation of transcription, DNA-templated	26	2.86E-02
	GO:1901701	cellular response to oxygen-containing compound	20	3.26E-02
	GO:0042593	glucose homeostasis	9	3.41E-02
	GO:1901700	response to oxygen-containing compound	25	3.63E-02
	GO:0048729	tissue morphogenesis	14	3.87E-02
	GO:0051254	positive regulation of RNA metabolic process	27	3.94E-02
	GO:1902680	positive regulation of RNA biosynthetic process	26	4.12E-02
	GO:0051240	positive regulation of multicellular organismal process	29	4.13E-02

	GO:0005488	binding	122	4.27E-02
	GO:1901699	cellular response to nitrogen compound	14	4.33E-02
	GO:1903508	positive regulation of nucleic acid-templated transcription	26	4.39E-02
	GO:0048568	embryonic organ development	12	4.69E-02
	GO:0045944	positive regulation of transcription by RNA polymerase II	21	5.00E-02
Pleiotropic	GO:0097110	scaffold protein binding	6	3.63E-06

Supplementary Table 8. Conjunction cFDR for 35 pleiotropic CpG-SNPs in FG and BW (cFDR < 0.05)

Variant	chr	P_FG	P_bw	Gene	SNP Type	cFDR_FG	cFDR_BW	ccFDR
rs1012635	6	5.13E-07	4.00E-10	CDKAL1	pleiotropic/confirmed	0.04886625	0.01573	0.04886625
rs1042725	12	0.002725	7.10E-32	HMGA2	T2D/confirmed	2.79E-06	0.000144	0.000144
rs1447351	11	2.83E-93	0.00023	MTNR1B	novel/confirmed	0.0291375	1.40E-09	0.0291375
rs151216	11	0.003477	5.10E-05	KCNQ1,KCNQ1OT1	novel/confirmed	9.54E-10	1.20E-18	9.54E-10
rs17361324	3	3.18E-10	8.00E-20	ADCY5	novel/confirmed	0.0081405	0.0077625	0.0081405
rs2206734	6	8.27E-08	2.40E-17	CDKAL1	novel/confirmed	2.99E-09	0.01993333	0.01993333
rs231354	11	5.94E-05	0.0028	KCNQ1,KCNQ1OT1	pleiotropic/confirmed	2.05E-06	4.00E-09	2.05E-06
rs340883	1	1.45E-09	0.0071	PROX1-AS1	T2D/confirmed	3.55E-07	0.01893333	0.01893333
rs4677887	3	0.019425	2.30E-12	ADCY5	pleiotropic/confirmed	1.65E-07	2.64E-16	1.65E-07
rs569255	3	0.000243	0.00045	SLC12A8	novel/confirmed	0.01957829	0.00526	0.01957829
rs579459	9	4.85E-05	8.30E-06	ABO	novel/confirmed	0.02516222	0.01199333	0.02516222
rs6006393	22	0.00034	0.0051	AC002378.1	novel/confirmed	0.01744615	0.02262308	0.02262308
rs6770420	3	8.30E-12	0.0046	KLF7P1	novel/confirmed	0.00070325	0.00014733	0.00070325
rs7004862	8	4.00E-04	0.0017	INTS8	novel/confirmed	0.04133767	0.00349633	0.04133767
rs7605661	2	0.002355	0.00039	THADA	novel/confirmed	0.00461538	0.02184	0.02184
rs7723	7	0.000926	0.00014	TMED4	T2D/confirmed	5.15E-91	0.00046	0.00046
rs7816345	8	0.000871	0.00042	AC090453.1	novel/confirmed	0.00545	3.71E-29	0.00545
rs9289218	3	1.13E-07	1.80E-05	ADCY5	novel/confirmed	0.0249475	0.049725	0.049725
rs16856159	2	7.51E-24	0.0068			5.35E-21	0.0204	0.0204
rs2001350	2	0.000191	0.0018			0.01005355	0.02061818	0.02061818
rs7748736	6	0.00108	0.0029			0.04764	0.04575556	0.04764
rs1059288	6	0.002113	0.00027			0.0431052	0.012096	0.0431052
rs4143341	7	0.001696	3.20E-06			0.018232	0.0003032	0.018232
rs11145756	9	2.85E-06	0.0015			0.00021375	0.00792857	0.00792857
rs3812605	9	0.000481	0.0042			0.02704289	0.04456667	0.04456667
rs17566087	10	0.003405	2.30E-05			0.03490125	0.00173938	0.03490125
rs198476	11	7.94E-05	0.00046			0.0026996	0.005175	0.005175
rs2072114	11	1.04E-15	0.003			4.27E-13	0.0165	0.0165
rs4601728	11	0.00039	0.0033			0.0213525	0.03526875	0.03526875
rs703545	12	0.007068	8.70E-06			0.04790533	0.00107107	0.04790533
rs7331478	13	2.85E-09	6.50E-05			1.66E-07	0.0005525	0.0005525
rs11074093	15	0.000352	0.00038			0.010912	0.007695	0.010912
rs4965425	15	0.006996	6.40E-07			0.0461736	0.00014029	0.0461736
rs2429243	17	4.37E-05	0.00092			0.00250339	0.00893714	0.00893714
rs6075924	20	0.003485	4.80E-06			0.0296225	0.0004944	0.0296225

Supplementary Table 9. Conjunction cFDR for 6 pleiotropic CpG-SNPs in FI and BW (cFDR < 0.05)

Variant	chr	P_FI	P_bw	SNP Type	Gene	cFDR_FI	cFDR_BW	ccFDR
rs9289218	3	0.000476	1.80E-05	novel/confirmed	ADCY5	0.017374	0.000792	0.017374
rs17361324	3	0.003383	8.00E-20	novel/confirmed	ADCY5	0.0050745	1.91E-17	0.0050745
rs900399	3	0.002526	2.90E-41			0.002526	1.06E-38	0.002526
rs2206734	6	0.010898	2.40E-17	novel/confirmed	CDKAL1	0.01453067	1.09E-14	0.01453067
rs7766106	6	3.08E-05	2.60E-05			0.0013398	0.000169	0.0013398
rs703545	12	1.52E-06	8.70E-06			9.12E-05	4.35E-05	9.12E-05

Supplementary Table 10. Conjunction cFDR for 54 pleiotropic CpG-SNPs in T2D and BW_maternal (cFDR < 0.05)

Variant	chr	P_DM	P_BW	Gene	SNP Type	cFDR_DM	cFDR_BW	ccFDR
rs1447351	11	6.60E-18	1.22E-08	MTNR1B	novel/confirmed	5.94E-17	2.68E-07	2.68E-07
rs1515114	2	9.50E-17	0.009981	AC062015.1	novel/confirmed	1.66E-14	0.027725	0.027725
rs17361324	3	1.10E-25	1.29E-05	ADCY5	novel/confirmed	2.97E-24	0.00016705	0.00016705
rs2206734	6	4.70E-66	7.74E-05	CDKAL1	novel/confirmed	2.73E-64	0.00023226	0.00023226
rs2488071	10	1.30E-22	0.0005663	Y_RNA	T2D/confirmed	8.36E-21	0.00302027	0.00302027
rs340883	1	1.40E-13	0.005544	PROX1-AS1	T3D/confirmed	1.36E-11	0.019404	0.019404
rs4677887	3	0.0011	0.0001349	ADCY5	pleiotropic/confirmed	0.0053625	0.00738578	0.00738578
rs9289218	3	3.60E-17	5.01E-16	ADCY5	novel/confirmed	3.60E-17	1.15E-14	1.15E-14
rs934227	2	1.40E-09	2.03E-05			1.01E-08	0.00027148	0.00027148
rs2580770	2	0.00021	0.003075			0.00434	0.04930603	0.04930603
rs175238	2	0.0011	7.63E-05			0.00447857	0.00476813	0.00476813
rs10190207	2	0.0036	0.0005613			0.02973913	0.03628927	0.03628927
rs12631028	3	2.00E-08	9.40E-06			1.30E-07	0.00021843	0.00021843
rs1826215	3	3.80E-05	1.76E-05			0.00014356	0.00056061	0.00056061
rs6440003	3	7.50E-07	2.51E-07			2.44E-06	8.90E-06	8.90E-06
rs3852060	3	1.60E-09	0.003731			1.06E-07	0.02374273	0.02374273
rs7657332	4	0.0027	0.00067			0.023004	0.0340896	0.0340896
rs4146009	4	0.0022	0.0007345			0.019536	0.03402204	0.03402204
rs6827183	4	0.0031	0.0001024			0.01298125	0.008736	0.01298125
rs7715701	5	0.017	4.16E-05			0.0499375	0.01007051	0.0499375
rs7701346	5	3.80E-07	8.81E-07			1.43E-06	2.95E-05	2.95E-05
rs1650504	5	1.90E-05	0.001422			0.00041563	0.02141888	0.02141888
rs2842363	6	1.80E-09	0.002199			7.95E-08	0.01459336	0.01459336
rs13220047	6	0.006	0.0004588			0.04175	0.03765983	0.04175
rs1415701	6	4.20E-05	0.0004987			0.000534	0.01040146	0.01040146
rs6918311	6	2.10E-11	0.01142			3.10E-09	0.04663167	0.04663167
rs2392244	7	0.0041	6.29E-05			0.01522857	0.00718179	0.01522857
rs1127065	7	2.20E-08	0.001289			6.52E-07	0.01113227	0.01113227
rs10758593	9	1.60E-10	8.98E-07			8.53E-10	1.68E-05	1.68E-05
rs2811709	9	3.40E-05	0.001777			0.00075337	0.02590679	0.02590679
rs2383208	9	2.60E-67	0.001181			4.00E-65	0.001181	0.001181
rs10983319	9	0.0021	0.0005646			0.01823182	0.02917955	0.02917955
rs7904519	10	1.70E-135	0.0006389			3.49E-133	0.0006389	0.0006389
rs1225404	10	2.80E-20	0.004352			3.80E-18	0.01450667	0.01450667
rs2334499	11	3.60E-05	0.001989			0.00081	0.0280449	0.0280449

rs2762954	11	8.90E-07	0.00199	2.51E-05	0.01815875	0.01815875
rs474901	11	0.0065	7.76E-06	0.0186875	0.00203383	0.0186875
rs7102746	11	2.80E-06	0.005975	0.00013185	0.0478	0.0478
rs12819124	12	2.20E-05	0.003385	0.0007403	0.0423125	0.0423125
rs1826535	12	4.50E-05	0.0003167	0.00047769	0.00725974	0.00725974
rs17331697	12	3.20E-05	0.0003115	0.00039855	0.00775918	0.00775918
rs6489844	12	0.00023	3.40E-06	0.00065714	0.00023911	0.00065714
rs3184504	12	2.30E-07	1.22E-14	2.30E-07	7.87E-13	2.30E-07
rs1054852	12	3.10E-09	0.005067	2.03E-07	0.02859236	0.02859236
rs10781628	12	0.00026	0.001917	0.00441	0.03782388	0.03782388
rs9593509	13	0.0051	5.03E-05	0.01748571	0.00652662	0.01748571
rs8022758	14	0.00021	2.56E-05	0.000665	0.00100092	0.00100092
rs12442879	15	9.20E-05	1.49E-05	0.00031689	0.00059931	0.00059931
rs7343010	18	1.80E-05	0.0003404	0.00023564	0.00736502	0.00736502
rs3803915	19	8.80E-05	0.00193	0.00169878	0.03004087	0.03004087
rs7248104	19	0.0073	4.52E-06	0.0191625	0.00125827	0.0191625
rs7246440	19	3.60E-05	0.002804	0.00090313	0.03474522	0.03474522
rs2304130	19	1.90E-15	0.001429	8.41E-14	0.00464425	0.00464425
rs2708742	19	0.00061	0.0002167	0.00431067	0.00980929	0.00980929

Supplementary Table 11. Conditional FDR value of 133 CpG-SNPs for BW_fetal given the DM (cFDR < 0.05)

Variant	Chr	Gene	Snp Type	P	cFDR
rs1012635	6	CDKAL1	confirmed/confirmed	3.27E-11	1.80E-10
rs1042725	12	HMGA2	novel/confirmed	6.72E-21	9.75E-20
rs10840346	11	SBF2	novel/confirmed	1.93E-06	0.00040448
rs10878353	12	HMGA2	novel/confirmed	1.17E-07	1.30E-05
rs10878359	12	MIR6074	novel/confirmed	6.02E-11	2.63E-08
rs11264298	1	DCST1	novel/confirmed	0.0002617	0.03691187
rs12306172	12	SMUG1,SMUG1-AS1	novel/confirmed	3.96E-06	0.00736571
rs12623454	2	AC073257.1	novel/confirmed	8.48E-06	0.00016646
rs12677785	8	SLC45A4	novel/confirmed	1.82E-05	0.0219353
rs12704091	7	ZNF783	novel/confirmed	6.46E-05	0.03083022
rs12865243	13	LINC00598	novel/confirmed	0.001429	0.03875112
rs12939237	17	IGF2BP1	novel/confirmed	6.15E-05	0.0040557
rs137848	22	IL17REL	novel/confirmed	8.56E-05	0.00066145
rs1415181	1	GAPDHP24	novel/confirmed	1.94E-05	0.02138222
rs151216	11	KCNQ1,KCNQ1OT1	novel/confirmed	0.0005676	0.00502108
rs1515114	2	AC062015.1	novel/confirmed	8.29E-06	2.96E-05
rs1547669	6	MLN	novel/confirmed	1.52E-06	0.001656
rs163177	11	KCNQ1	novel/confirmed	0.003422	0.013688
rs17361324	3	ADCY5	novel/confirmed	2.80E-26	3.64E-25
rs17401675	7	MLXIPL	novel/confirmed	5.34E-10	6.74E-08
rs17689040	7	SUGCT	novel/confirmed	0.0003883	0.02320925
rs1886843	20	STX16,STX16-NPEPL1	novel/confirmed	6.68E-06	0.00281015
rs2000244	9	STRBP	novel/confirmed	2.20E-06	7.30E-05
rs2206734	6	CDKAL1	novel/confirmed	1.86E-19	5.57E-19
rs2236407	9	PTCH1	novel/confirmed	1.57E-07	1.62E-05
rs2426778	20	NPEPL1	novel/confirmed	6.77E-05	0.04231615
rs2488071	10	Y_RNA	novel/confirmed	4.86E-09	1.56E-08
rs2745929	6	CDKAL1	novel/confirmed	1.39E-09	5.55E-09
rs2886070	1	ARHGEF2	novel/confirmed	3.83E-07	0.00090853
rs2894593	2	AC062015.1	novel/confirmed	0.001014	0.002873
rs3213225	11	IGF2,INS-IGF2	novel/confirmed	2.82E-10	1.88E-08
rs340883	1	PROX1-AS1	novel/confirmed	0.0003724	0.00144822
rs390200	17	DLG4	novel/confirmed	2.37E-08	3.67E-05
rs452674	13	LINC00598	novel/confirmed	0.0002213	0.01337943
rs4625714	16	LPCAT2	novel/confirmed	7.11E-05	0.02681838
rs4677887	3	ADCY5	confirmed/confirmed	0.0003492	0.01456663
rs4677889	3	ADCY5	novel/confirmed	1.05E-05	0.00147809
rs492602	19	FUT2	novel/confirmed	6.51E-06	0.01085498
rs4965425	15	AC118658.1	novel/confirmed	1.76E-06	0.00393077
rs4980661	11	AP000439.2	novel/confirmed	9.89E-06	0.00659886
rs6016377	20	MAFB	confirmed/confirmed	1.41E-08	2.30E-05
rs6057610	20	C20orf203	novel/confirmed	1.05E-09	8.45E-07
rs6075924	20	LINC00261	novel/confirmed	7.58E-06	0.00628746
rs6770420	3	KLF7P1	novel/confirmed	0.002812	0.00723086
rs7004862	8	INTS8	novel/confirmed	0.001778	0.01012092

rs7088711	10	WBP1L	novel/confirmed	9.90E-05	0.02317354
rs7331478	13	PDX1	novel/confirmed	8.00E-06	0.00719778
rs7561273	2	MFSD2B	novel/confirmed	5.88E-06	0.01162883
rs7605661	2	THADA	novel/confirmed	0.007443	0.0302682
rs7766106	6	RSPO3	novel/confirmed	0.0005544	0.0422037
rs7816345	8	AC090453.1	novel/confirmed	0.002037	0.03783
rs7965495	12	RPL21P18	novel/confirmed	3.64E-06	0.00010424
rs8182579	19	PEPD	novel/confirmed	0.0001873	0.00181057
rs855715	10	ADRB1	novel/confirmed	1.12E-05	0.00390828
rs900399	3	LINC00880	novel/confirmed	1.55E-37	5.44E-34
rs926345	20	PLCG1	novel/confirmed	1.36E-05	0.00091218
rs9289218	3	ADCY5	novel/confirmed	9.89E-17	7.58E-16
rs935172	2	CIB4	novel/confirmed	0.0001418	0.01920556
rs9416062	10	LRMDA	novel/confirmed	0.007054	0.04856408
rs4655772	1			0.0004808	0.04171463
rs17407594	1			1.91E-05	0.00838113
rs2884428	1			9.98E-07	0.00240745
rs13020622	2			1.08E-05	0.00099688
rs10865186	2			0.0001158	0.03633099
rs895514	2			0.0002029	0.02075471
rs6749108	2			0.0003793	0.01269657
rs4664054	2			0.0001046	0.04485819
rs853770	2			1.78E-06	0.00387095
rs16856159	2			5.42E-05	0.02007762
rs1801123	2			0.0003899	0.03399758
rs332353	3			0.003545	0.0328976
rs13075511	3			0.0006302	0.018906
rs3852060	3			0.002468	0.01328923
rs7657332	4			2.54E-05	0.00170314
rs1460554	4			0.0008588	0.03377947
rs7666523	4			2.06E-05	0.01891119
rs10512645	5			1.40E-05	0.0278654
rs12658884	5			1.66E-05	0.02684736
rs13180312	5			0.0001105	0.03770582
rs17056278	5			0.0002935	0.03992283
rs16884481	6			0.001217	0.02822281
rs1466339	6			0.004848	0.02477867
rs9368716	6			0.00705	0.048222
rs13219530	6			0.0001734	0.038135
rs4236049	6			0.0002469	0.04212803
rs13220047	6			0.0007954	0.04123521
rs1262557	6			3.13E-06	1.72E-05
rs9457107	6			1.07E-05	0.01209444
rs2934849	6			6.75E-05	0.02081225
rs38205	7			0.001059	0.024357
rs6948977	7			0.0002126	0.03499508
rs2392244	7			0.0001059	0.00736235
rs1127065	7			0.002815	0.01573088

rs10265057	7	9.28E-07	0.00042795
rs42042	7	1.48E-05	0.00648605
rs10487687	7	7.50E-05	0.04762364
rs7016707	8	0.006436	0.04994336
rs551580	8	3.07E-05	0.00695603
rs13255921	8	0.0005765	0.02419165
rs7460241	8	1.80E-05	0.02264809
rs10758593	9	6.16E-08	4.31E-07
rs10992100	9	6.24E-05	0.03037422
rs357542	9	2.77E-05	0.00127327
rs17301196	9	0.0002614	0.01143281
rs11145846	9	0.003154	0.0184509
rs11145756	9	0.001304	0.010758
rs3812605	9	0.0009563	0.02798217
rs11009689	10	0.001576	0.03594297
rs2782979	10	0.00143	0.02403762
rs2292623	10	0.003152	0.04919371
rs3213223	11	3.84E-06	0.00072623
rs7483056	11	7.28E-05	0.01885261
rs9669403	12	0.000226	0.00652741
rs10878361	12	3.41E-05	0.00500507
rs7336104	13	0.0001887	0.0145299
rs9593509	13	0.000256	0.01604855
rs4899027	14	0.0002498	0.01131988
rs8022758	14	0.000625	0.01542763
rs11160605	14	1.65E-05	0.00235233
rs12442879	15	0.002313	0.03640461
rs7178220	15	1.82E-05	0.00565695
rs208569	16	0.0002637	0.04940443
rs13336802	16	8.31E-05	0.02231026
rs1242507	17	0.0003098	0.02684662
rs7220080	17	2.36E-06	0.00239079
rs11658711	17	0.0001492	0.03589127
rs7248104	19	3.01E-05	0.00305272
rs3786913	19	0.001317	0.01293759
rs2303088	19	3.83E-05	0.03451953
rs205894	20	0.0001576	0.04472151
rs6119294	20	2.06E-06	0.00367933
rs2766673	20	0.002079	0.04934889
rs911303	20	0.008403	0.02881029
